# The Mental Maxwell Relations: A Thermodynamic Allegory for Higher Brain Functions

**DOI:** 10.3389/fnins.2022.827888

**Published:** 2022-02-28

**Authors:** Joseph O’Neill, Andreas Schoth

**Affiliations:** ^1^Division of Child and Adolescent Psychiatry, UCLA Semel Institute for Neuroscience, Los Angeles, CA, United States; ^2^IMTEK Department for Process Technology, Institute of Microsystem Technology, Universität Freiburg, Freiburg, Germany

**Keywords:** consciousness, volition, information, attention, salience, arousal, distraction

## Abstract

The theoretical framework of classical thermodynamics unifies vastly diverse natural phenomena and captures once-elusive effects in concrete terms. Neuroscience confronts equally varied, equally ineffable phenomena in the mental realm, but has yet to unite or to apprehend them rigorously, perhaps due to an insufficient theoretical framework. The terms for mental phenomena, the mental variables, typically used in neuroscience are overly numerous and imprecise. Unlike in thermodynamics or other branches of physics, in neuroscience, there are no core mental variables from which all others formally derive and it is unclear which variables are distinct and which overlap. This may be due to the nature of mental variables themselves. Unlike the variables of physics, perhaps they cannot be interpreted as composites of a small number of axioms. However, it is well worth exploring if they can, as that would allow more parsimonious theories of higher brain function. Here we offer a theoretical exercise in the spirit of the National Institutes of Health Research Domain Criteria (NIH RDoC) Initiative and the Cognitive Atlas Project, which aim to remedy this state of affairs. Imitating classical thermodynamics, we construct a formal framework for mental variables, an extended analogy – an allegory – between mental and thermodynamic quantities. Starting with mental correlates of the physical indefinables length, time, mass or force, and charge, we pursue the allegory up to mental versions of the thermodynamic Maxwell Relations. The Maxwell Relations interrelate the thermodynamic quantities *volume*, *pressure*, *temperature*, and *entropy* and were chosen since they are easy to derive, yet capable of generating nontrivial, nonobvious predictions. Our “Mental Maxwell Relations” interlink the mental variables consciousness, salience, arousal, and distraction and make nontrivial, nonobvious statements about mental phenomena. The mental system thus constructed is internally consistent, in harmony with introspection, and respects the RDoC criteria of employing only psychologically valid constructs with some evidence of a brain basis. We briefly apply these concepts to the problem of decision-making and sketch how some of them might be tested empirically.

*By a physical analogy I mean that partial similarity between the laws of one science and those of another which makes each of them illustrate the other.*

Maxwell (1855)

*(Because of the weakness of the human brain, we can’t think of something really new, so we argue by analogy with what we know).*

Feynman et al. (1964)

## Introduction

Have you ever noticed the variables used in neuroscience for higher brain functions are often inelegantly numerous and imprecise? Sampling recent fMRI papers finds “reversal-learning, tactile sensation, hallucinations, mental rotation, social hierarchy, orienting, intentionality, syntactic processing,…” Other terms, e.g., “information, activity, integration, representation, processing, encoding, regulation,…,” are ubiquitous but rarely defined. Which terms designate *bona fide* brain or mind entities and which are mere operational labels? Which are separate, overlapping, derivative? Empiricism is a neuroscience virtue, but promotes this prolific, indeterminate vocabulary. That is, it is safer to label distinct phenomena than to generalize even modestly. This lack of theory may hinder the field in relating mental phenomena to the physical world. For theory can organize and systematize phenomena, even without new data. Hence, there have been recent calls for more theory in biology generally (Nurse, 2021). Within neuroscience, the Cognitive Atlas Project (Bilder et al., 2009; Miller et al., 2010; Poldrack et al., 2011) strives for an atlas of latent cognitive constructs to build a “map of the mind” (heterophenomenology, introspection; James, 1890; Dennett, 1991) to promote clarity in a field fraught with ambiguous and misused and/or misunderstood terms. The National Institutes of Mental Health (NIMH) Research Domain Criteria (RDoC) initiative (Insel et al., 2010; Morris and Cuthbert, 2012) is a comparable system-building effort to establish a comprehensive set of valid constructs linked to neural circuits and behavior. Inspired by these projects, we aim here to erect an internally coherent system of mental constructs for eventual external validation. Each construct should be in harmony with introspection and have an at least tentative brain basis.

Our strategy appeals to classical thermodynamics. We are not investigating brain thermodynamics *per se*; others (e.g., Deli et al., 2021) are ably pursuing that goal. Rather, we borrow the mathematical formalism of thermodynamics to create an analogous formalism for higher brain functions. A similar approach was taken by Khrennikov (2006, 2011) using quantum mechanics. Quantum mechanics may be closer to actual brain processes, but, to confine the scope, we have largely eschewed quantum mechanics in the present paper. The present treatment, however, may be seen as establishing a classical-physics analogy to serve as a foundation for future quantum-inspired modeling efforts. Since ours is a complex, multistep analogy, we call it an *allegory*, an extended analogy. Nineteenth-century thermodynamics resembled cognitive neuroscience today. It was recognized that objects had thermodynamic properties such as volume, pressure, and temperature, but it was not known how these properties interrelated. There was an intuitive sense of “heat” and “work,” but it was uncertain how to calculate work and whether heat was a substance or a property. Theories of Maxwell, Boltzmann, Clausius, and others helped clarify these issues. Quantitative definitions of variables rooted in mechanics were postulated, and their interrelations were worked out. Some terms (heat, work) were redefined, new terms (enthalpy, entropy) were introduced, and old terms (phlogiston, caloric, frigoric) were abandoned. Perhaps one can likewise propose a set of more useful mental concepts with common axiomatic sources and clear interrelations. Along the way, terminology may be clarified, alleviating the reigning confusing proliferation of endpoints.

Here we make a modest effort in that regard, a mathematical exercise centering on the *Maxwell Relations*, four equations interconnecting the thermodynamic variables *volume*, *pressure*, *temperature*, and *entropy*. Below we propose *consciousness*, *attention*, *arousal*, and *distraction* as mental variables analogous to these four that form a set of “Mental Maxwell Relations (MMR),” concerning mainly conscious brain processes. We focus on the Maxwell Relations because they are simple, yet allow nontrivial, nonobvious predictions. Maxwell employed different varieties of analogy in his theoretical development (Achinstein, 2013). Some were pure analogies like the planetary model of the Rutherford atom or Baars’s (1998) stage metaphor for consciousness that give a picture without providing a causal explanation. Others incorporated physical aspects of the system being modeled more directly. His analogies were characteristically worked out in great detail. Thereby, he mixed preexisting empirical evidence with (at the time) unproven or nonexistent entities (e.g., molecules, an imaginary fluid that models electricity) and included both testable and (at the time) not-testable assumptions and predictions. The present allegory is largely model construction – “an exercise in mechanics” to use Maxwell’s phrase – that explores parallel mathematical structures. But where we see points of contact [e.g., consciousness as an indefinable of physics, see “Mental Consciousness ⇔ Physical Volume”; the Equation for Consciousness, (Eq. 6)] between thermodynamics and higher brain function we do make analogic inferences (Achinstein, 2013), when we suspect they may yield insights into the workings of mind and brain. Some MMR predictions might later be tested experimentally to determine whether the mental-thermodynamic allegory has traction. However, for now the task is merely to set up the formalism. The goal of this manuscript is simply to see how far the allegory can be taken without losing coherence or introspective validity. Starting with the core indefinables of physics and continuing to thermodynamics, we propose a mental analog for each physical variable. We explain how each choice is introspectively appealing, sometimes making intermediate observations. Then, respecting RDoC rules, we cite work supporting the mental variable as a psychologically valid construct with a brain basis. We use our mental variables to formulate the MMR. We suggest a few MMR predictions and a few ways to test our formalism experimentally. We show how these mental variables might combine to generate behavior in a “Kant Cycle” (perception–cognition–emotion–behavior cycle) analogous to the thermodynamic Otto Cycle, and we discuss the relevance of MM to decision-making.

## The Thermodynamic Maxwell Relations

The Maxwell Relations (Eq. 1) interrelate volume, pressure, temperature, and entropy (*V*, *P*, *T*, *S*) of a thermodynamic system. An advanced version (Eq. 2) replaces *P* and *V* with the *stress tensor*, σ, and the natural (Hencky) *strain tensor*, ε, times reference volume, *V*_0_.

The basic **Thermodynamic Maxwell Relations** are


(1) $$\begin{array}{c} {\left(\frac{\partial T}{\partial V}\right)}_{S}=-{\left(\frac{\partial P}{\partial S}\right)}_{V},{\left(\frac{\partial T}{\partial P}\right)}_{S}={\left(\frac{\partial V}{\partial S}\right)}_{P}\\ {\left(\frac{\partial V}{\partial T}\right)}_{P}=-{\left(\frac{\partial S}{\partial P}\right)}_{T},{\left(\frac{\partial P}{\partial T}\right)}_{V}={\left(\frac{\partial S}{\partial V}\right)}_{T}. \end{array}$$

In advanced form they are written


(2) $$\begin{array}{c} \;{\left(\frac{\partial T}{\partial \varepsilon V}\right)}_{S,n_{i}}=-{\left(\frac{\partial \sigma }{\partial S}\right)}_{\varepsilon V_{0},n_{i}},\\ \;{\left(\frac{\partial T}{\partial \sigma }\right)}_{S,n_{i}}={\left(\frac{\partial \varepsilon V_{0}}{\partial S}\right)}_{\sigma ,n_{i}}\\ {\left(\frac{\partial \varepsilon V_{0}}{\partial T}\right)}_{\sigma ,n_{i}}=-{\left(\frac{\partial S}{\partial \sigma }\right)}_{T,n_{i}},\\ \;{\left(\frac{\partial \sigma }{\partial T}\right)}_{\varepsilon V_{0},n_{i}}={\left(\frac{\partial S}{\partial \varepsilon V}\right)}_{T,n_{i}} \end{array}$$

For multicomponent mixtures, further Relations accrue involving the number of moles, *n_i_*, and chemical potential, μ_*i*_, of each component. MM correlates of *n_i_* and μ_*i*_ are discussed in the Supplementary Material. The reciprocal of each Relation forms an additional Relation. These equations make nontrivial, non-intuitive predictions. Take, for example, a blob of gelatin as thermodynamic system. The third Maxwell Relation says the change in gel volume per change in temperature at constant pressure (subscript *P*) equals minus the change in entropy of the gel with change in pressure at constant temperature. Intuitively (most) substances increase in volume with temperature at constant pressure (left-hand side of equation). However, it is *not* obvious that the substance entropy decreases with pressure at constant temperature (right-hand side) or that the two rates of change are equal. However, the Maxwell Relations assure us they are and conveniently allow us to *calculate* things difficult to measure. Suppose, for example, you wish to know how the entropy of a fixed volume of gel changes with pressure. That is challenging to measure. How do you quantify entropy? Can you keep the gel volume from changing when you apply pressure or vacuum to it? Fortunately, the first reciprocal Maxwell Relation tells you that, if you measure the rate of change of gel volume with temperature (e.g., by pouring it into a graduated cylinder and heating) at constant entropy (i.e., heating reversibly – slowly and gently), that gives you minus the rate of change of entropy with pressure at constant volume. The Maxwell Relations enable such shortcuts and workarounds. Experimental verification of unexpected predictions of the Maxwell Relations has reinforced the validity of the thermodynamic formalism overall. If validity can one day be shown for the MMR or similar equations, such shortcuts and workarounds may become possible in cognitive neuroscience.

## Mental Analogs of Core Physical Variables

### Number and Information

Our allegory begins with information, the most overused, underdefined term in neuroscience where it may refer to semantic meaning, patterns of neural activity, values of biophysical parameters, etc. Information in the MM system is the Shannon (1948) bit, derived from the concept of number.

#### Number

The MM definition of a (natural) number is: “A universal symbol for denoting elements of sets that combines the cardinal and the ordinal property. The cardinal property means treating different things alike; the ordinal property means treating alike things differently.” Imagine counting cabinet knobs on a display. One is wood, one brass, one obsidian,…; one is round, one square, one triangular,…; the fasteners vary, etc. Despite wide differences, each knob counts as “1.” This is the cardinal property: details present in an object are abstracted away, reducing it to a number. We are treating different things alike. Now imagine you are sorting a bag of effectively identical knobs to ship each to a different store. You label each, mentally or physically, “1, 2, 3,….” This is the ordinal property: details (the labels) not present are added to the objects. We are treating alike things differently. There are rules: you may not double-count or double-back on the order, nor skip any member of the set. These rules are easily communicated and agreed upon among human beings. In keeping them, you enforce an objectivity that closely models the indifference of nature to our personal concerns. The twin paradoxes of cardinality and ordinality underlie most mathematics. Their flexibility allows mathematics to function at any level of approximation and to deal with the actual, the possible, and the impossible. The brain clearly shares this ability to treat different things alike and to treat alike things differently and, hence, functions as much by truth as by falsity. For example, if you hear rustling in the bush, it is not yet a fact that it is a leopard – that is only a probability or possibility. Yet, it is potentially life saving to imagine it is a leopard, to insert a fact not in evidence. In such manner, the observed and the conjectured intermix regularly in mental life as a practical necessity.

Cardinal and ordinal numbers are recognized cognitive constructs with apparent brain bases in frontal and parietal cortices, particularly the inferior parietal sulci (Nieder and Dehaene, 2009) and, thus, satisfy RDoC criteria.

#### Mental Information ⇔ Physical Energy

*Energy* results when force is applied to mass over distance. *Information*, the MM correlate of energy, results when attention (force correlate; see “Mental Attention ⇔ Physical Force”) is applied to a concept (mass correlate; see “Mental Inertia, Concept ⇔ Physical Mass, Particle”) in consciousness (space correlate; see “Mental Consciousness ⇔ Physical Volume”). Like cardinal numbers, energy is universal: it is stored in numerous distinct modes and flows fungibly between them. Information in MM is also cardinal: it is the *quantity* of each sensory quality, or *attribute* (see “Mental Consciousness ⇔ Physical Volume”), experienced momentarily in consciousness. It is the number of *countable* but not distinguishable units experienced in each attribute. The attributes information is stored in and flows between are numerous.

The MM unit of information is 1 bit. A bit is like a number. It is the resolution, the smallest difference between two states or objects an observer or instrument can or opts to distinguish. Just as all features are discarded when counting objects, reducing them to numbers, so is the inside of a bit featureless; one treats it as a pure number. Moreover, like numbers, bits are universal. The validity of MM information rests on its identity with Shannon information. We cite no brain center or network as the basis of information since it inheres throughout the brain.

### Mental Time ⇔ Physical Time

*Time*, like length, mass or force, and charge, is a fundamental indefinable of physics, a measurable, but not definable quantity. The MM correlate of physical time is mental *time*. Time unites the physical and mental realms, although mental time is distorted relative to physical, laboratory time. Like number, physical time and mental time are universal; all events are assigned a timepoint regardless of their character. Time is like a symbol, void of content, in that all details of an event are abstracted away in assigning its timepoint. Time is the one thing that changes if everything else remains the same. Time is cardinal (duration) and ordinal (arrow of time). In measuring time, one may not double-count, double-back, or skip any interval.

The MM unit for mental time is the 200 ± 30-ms mean interval between ocular saccades (“1 saccade”). Saccades are the frequent jumps lasting 20–200 ms the human eye makes from feature to feature in beholding a scene (Saslow, 1967). The retinae are fixed during the intersaccade interval while the brain constructs a mental image to be replaced upon the next saccade. This time-chunking structures not only vision but also other modalities of consciousness. In muscle contraction, the binding period for myosin to actin is also 200 ms (Alberts et al., 1983) and motor action supports all senses (e.g., eye movements, moving the fingertips). Libet’s (2004) 150–200-ms period during which a human can still “veto” an incipient voluntary movement is also ∼200 ms. This is also the period of the hippocampal θ-rhythm of long-term potentiation (LTP), a molecular mechanism of memory (Hyman et al., 2003). This common timing of eye movements, muscle contraction, response inhibition, memory formation, and consciousness refresh embodies efficient physiology. Mental time is established in cognitive phenomenology with brain bases imputed in the cerebellum, striatum, and supplementary and pre-supplementary motor cortices (Coull et al., 2011).

### Consciousness, Mental Velocity, Mental Acceleration, Mental Jerk

#### Mental Consciousness ⇔ Physical Volume

*Space* (length, area, or volume) is the venue of physical events. *Consciousness*, the MM correlate for space, is the venue of mental events. Once thought a pure void, space actually has properties including capacitance (electrical permittivity, ε_0_) and inductance (magnetic permeability, μ_0_) per unit length. Maxwell’s most celebrated achievement was to combine these into $c_{0}=\frac{1}{\sqrt{\varepsilon_{0}\mu_{0}}}$, the maximum transfer rate for matter, energy, or information across vacuum. In MM, consciousness, similarly, has a maximum information transfer rate, *c_M_* (see “Mental Velocity, Mental Acceleration, Mental Jerk”). Space influences material events by impeding transport, rendering it non-instantaneous. Consciousness may likewise interact with material events by impeding information transport in the brain. Having *c*_0_ < ∞ gives rise to Relativity and structures the world into space–time blocks. Having *c*_*M*_ < ∞ structures the mind into consciousness-time blocks.

The MM consciousness construct endorses Monti et al.’s (2009) division of conscious phenomena into three dimensions but uses diverse terminology. What we call “consciousness” is Monti’s first dimension “*content* of consciousness,” subjective “awareness.” His second dimension, “level of consciousness,” is “arousal” in MM (see “Mental Arousal ⇔ Thermodynamic Temperature”). His third dimension “ability to produce voluntary behavior, mobility” in MM is a combination of “will” (see “Mental Will, Emotion, Pain, Drives ⇔ Physical Yank”) plus an intact motor nervous system. Thus, MM consciousness is the ensemble of subjective features or qualities (“qualia”; Peirce, 1866) in the mind at the moment. MM calls them *attributes*.

MM rejects the materialist position that this subjective consciousness is reducible to the four indefinables of physics. Instead, we consider consciousness a *fifth* indefinable needed to measure the other four. Typically, materialism demands that all genuine phenomena be *measurable*. However, any physical measurement requires a *coordinate system* of some kind, be it as simple as a ruler, stopwatch, or thermometer. In MM, consciousness *is* the coordinate system; it assays the quantity of any physical variable during measurement or any sensory or imagined quality in lived experience generally. Consciousness is necessary for a full picture of the world including both physical quantities and the primary data (James, 1890) of subjective experience. If you doubt that coordinate systems in physics are essentially *mental*, think of metering a length of cloth against an old yardstick. Wherever a hash mark on the stick is effaced by wear, one reinserts it – in the mind – to bridge the gap and take the measure. Experimentalists routinely make such mental adjustments, even with highly sophisticated apparatus. Now, when we say consciousness is a coordinate system we do not mean there is a graduated gridwork of fine lines inside our heads, obviously there is not. Or that consciousness is only present during formal physical measurements, an infrequent, specialized activity. We mean that consciousness assesses the quantity of attributes of numerous types during behavioral experience of many kinds. Consciousness is the (usually informal, imprecise) moment-to-moment coordinate system the brain uses to span physical and mental events.

**FIGURE 1:**
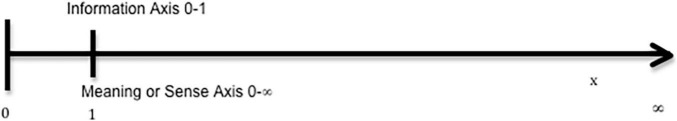
Consciousness, meaning, and information. *Consciousness* in the Mental Maxwell model is a set of coordinate axes, each metering the amount of a different sensory quality, or *attribute*. An axis is shown for attribute *x*. The axis is oriented; it runs from a starting point (0) to a destination (∞, or some finite maximum). This gives it *sense* or *meaning*. In going from origin to endpoint, one takes steps of a fixed size (1). *Information* is the number of these steps currently populating the axis, the value of the attribute. Note that information itself can be regarded as a minimalist axis (a bit-axis) where ∞ and 1 collapse leaving only 0 and 1. Thus, information alone has no sense and can enumerate any attribute. Alternatively, were consciousness maximized by breaking it up into bit axes (as one tends to do in meditation), it would ultimately degenerate into pure information.

Like any coordinate system, consciousness resembles a physical space. Consciousness in MM is a set of coordinate axes, one for each sensory quality, or attribute, experienced subjectively at a given moment. Each axis consists of a “0,” a “1,” and an “∞” (Figure 1). The 0 (origin) is a place to start, indicating none (or some reference amount) of the attribute is present in consciousness. The ∞ (or maximum) is a place to go, perhaps the greatest amount of the attribute (e.g., brightest light) manifestable in consciousness. The 1 (scale) is the size of the steps to take along the way, perhaps the resolution of the attribute (e.g., lightest weight). Information is the quantity of each attribute experienced in consciousness: it is the 1-units that populate the consciousness axes. Hence, consciousness is ordinal: it is the number of *distinguishable* categories experienced, while information is cardinal, the number of units in each category.

Consciousness in MM has not three, but *N* dimensions one for each attribute experienced at the moment. The axes of consciousness express such qualities as colors, visual and tactile shapes, tactile and visual textures, hot and cold, audio pitches, and more complex sensory dimensions. The three dimensions of physical space are included, albeit in perceptually distorted forms. There is an axis for each attribute in consciousness with a 0 and an ∞ and populated with 1’s (information) in between. Everyday conscious perception has numerous inborn and learned “bottom-up” scales and axes. Affect and cognition use fewer conscious axes. Note that everyday behavior frequently employs imprecise but functional “natural scales,” like the handful, the pace, the heartbeat,… Such scales enable us to judge that a stone lies within grasp, the horizon is far to run, it is nearly sunset, etc. Thus, consciousness is an information space, behaviorally scaled. We refer to the full set of axes as a mental *scene*. We use the term *context* for consciousness-time, a scene plus memory of past scenes.

For the MM units for consciousness, consider that physical space has *length*, *area*, and *volume*. The mental correlate of length has the unit 1 attribute and is the number of mental dimensions holding 1 bit of information, *N*. For the volume correlate, recall that volume, in statistical thermodynamics, means the number of space elements a particle can occupy within the system. The MM analog of a physical particle is a concept (see “Mental Inertia, Concept ⇔ Physical Mass, Particle”). Concepts are formed in MM by fusing multiple occupied attribute axes. We estimate the volume of an *N*-axis consciousness coordinate system as *N*!, the number of permutations of the axes, since this gives an indication of the number of different ways of chaining attributes together to compose concepts (actually, this is an underestimate, but we leave the more detailed computation aside). Its units are attribute^*N*^. We call the mental correlate of area the *background* and estimate it as (*N*−1)!, the permutations of all axes, except the one in focus at the moment. Its units are attribute^*N*−1^. If, for example, you focus on the green color of a leaf, then all its other attributes (shape, size, texture, etc.) and everything else in the scene is the background. An attribute (mental degree-of-freedom) is a distinguishable depot or mode to store or express information. Consciousness is a long-valid construct in psychology (e.g., Lashley, 1923). Several brain bases have been proposed (e.g., Newman, 1995; Smythies, 1997). Following Edelman et al. (2011), we surmise that contents of consciousness (attributes, qualia) arise through reentrant thalamocortical innervation.

#### Matrix Representation of Consciousness; Consciousness Deformation ⇔ Physical Strain

An *N*×*N matrix* is an alternative representation of a conscious coordinate system. The *N* matrix eigenvectors correspond to the coordinate axes; the *N* eigenvalues to their scalings. This formulation may help address the question, how can independent observers – each insulated in his or her own mind – share the same *subjective* meaning, of a word, phrase, action, etc.? Context or meaning might be shared between observers when their consciousness matrices are *similar*, i.e., have the same eigenvalues, when agreement and counting are possible due to a shared lowest resolution.

Matrices are operators, as in group theory. The number of consciousness axes, their orientations and scalings, etc., need not remain identical from moment to moment. Instead, moment-to-moment shifts in consciousness may be seen as a chain of multiplications by operators that compress, expand, rotate, bend, and rescale the axes of each mental scene to warp it into the next. Moreover, each successive scene is a translation of the scene midpoint along the subject’s episodic time axis. For a more general formulation that includes warpings of conscious dimensions, MM offers *consciousness deformation* as a mental correlate of the strain tensor in physics. Like the strain tensor, the diagonal elements of the consciousness-deformation tensor are dimensionless and indicate expansion and compression of each axis. The off-diagonal elements (*angular deformations* in physics) indicate the dimensionless fractional transformation of each axis into each of the others during shifts in consciousness. Roy and Llínas (2008) have previously developed a tensor model of consciousness.

#### Mental Velocity, Mental Acceleration, Mental Jerk

As physical *velocity* is length per unit time, its MM analog *mental velocity* is consciousness creation or destruction per unit time in attribute/saccade. Mental velocity evaluates how rapidly attribute axes are erected or torn down in consciousness, as a scene forms or disappears. In physical Special Relativity, *c*_0_ is the fastest possible velocity. Mental scenes likewise cannot change infinitely fast. To reflect this, MM assigns an upper limit to mental velocity, *c_M_*. We do not know what this limit is, but guess *c*_*M*_ = 7 ± 2 attribute/saccade, adapted from Miller (1956). Thus, mental, like physical, space–time has a finite slope. Using the MM constructs for information and association (see “Mental Information ⇔ Physical Energy” and “Mental Learning, Habit, Association ⇔ Physical Linear Momentum”), *c*_*M*_ = 7 ± 2 bit/link is alternatively the maximum information content of a mental association.

Mental velocity constructs are not widely used in neuroscience, but have been employed. D’Ercole et al. (2010), for example, speak of image formation speed as the time to generate a mental image. Kosslyn (1984) indicates that mental representation is voluntary, and the more complex an image, the more time it takes to form. This is consistent with our concept of attributes assembled at finite speed to generate a conscious scene. The MM formalism further allows analogs to physical *acceleration* (second time derivative of position) and *jerk* (third time-derivative): *mental acceleration*, the second time derivative of consciousness, and *mental jerk*, the third time derivative.

### Concept, Association, Attention

#### Mental Inertia, Concept ⇔ Physical Mass, Particle

*Mass* is the tendency of a body to stay together, to stay on trajectory, to resist changes in motion. It is quantized as *particles*. *Mental inertia* is the MM correlate of mass, quantized as *concepts*. With concepts in MM, we mean such entities as individual words, sounds, objects, actions, etc., as they manifest momentarily in consciousness. Mental inertia is the tendency of the diverse attributes of a concept to stay and move together as a single entity in consciousness. At any instant, a particle has a velocity and a location in space. Location gives it *potential energy*; velocity *kinetic energy*. At any instant, a concept has coordinates in the attribute space of consciousness; its velocity is how rapidly that attribute is appearing or disappearing from consciousness. The *potential information* of the concept is information it holds by virtue of its position in memory; the *kinetic information* of the concept is the information it draws out of (or carries into) memory. A particle travels through space–time as a compact *bundle* of modes of energy. A polyatomic molecule, for example, has distinguishable translational, rotational, vibrational, etc., modes for storing energy. A concept evolves through consciousness as a bundle of information in different modes. Suppose, for example, the concept is one leaf, on one bough of one tree we are looking at in the backyard. Looking at the leaf, our attention is guided to its outline. We perceive it as a single entity. It requires mental effort to stare, for example, not at the leaf, but at a featureless patch of sky to its right. It requires effort to stare, not at the outline of the leaf, but at its interior, to perceive its green, say, without its other attributes. Or to zoom out from the leaf and perceive the entire bough as a fuzzy green blob. To do this, one must overcome the mental inertia that creates the contours. Besides some amount of green, the leaf has so much yellow, so much roundness, so much roughness,… These attributes locate the leaf in the mental coordinate system of consciousness. Information is the amount of each quality manifested by the leaf at the moment in consciousness. The leaf, or any concept, is a carrier for modes of information. It is essentially the modes (category axes) being carried since the amount of information they contain goes up and down as the reflected light, the twist of the leaf in the wind, etc., shifts from moment to moment with the scene. Yet, we retain an impression of it being the same leaf; mental inertia is the tendency of a concept to stay together. Mental inertia carries a concept through to the next scene. In the famous rabbit–duck illusion (Wittgenstein, 1958), for example, once you have seen the rabbit or the duck, it is difficult to unsee them. This is analogous to a mass in physics swiftly shifting between spin-states. However, it is easy to put a random assemblage of lines out of mind. It has little staying power, low mental inertia.

Like physical particles, concepts are unitless; one simply counts the number present. Mental inertia is quantified in MM by analogy with mass–energy equivalence $E=mc_{0}^{2}$ in Relativity (in MM, the symbol for each mental variable results from affixing subscript *M* to the correspoonding physical variable). The information, *E_M_*, of a concept is the sum of the bits on all its attribute axes. Its mental inertia is $m_{M}=E_{M}/c_{M}^{2}$ with unit 1 bit ⋅ saccade^2^/attribute^2^ = 1 *link*/(attribute/saccade). Mass in mechanics is, similarly, energy gradient per unit acceleration or momentum per unit velocity. Thus, mental inertia is the time rate of change of consciousness required to latch onto or to break free from an association. Concepts are a regular topic in cognitive neuroscience (Antonucci and Alt, 2011). Concept formation may occur in the hippocampus, amygdala, or entorhinal cortex (Quiroga, 2012).

#### Concepts and Consciousness Interacting

**FIGURE 2:**
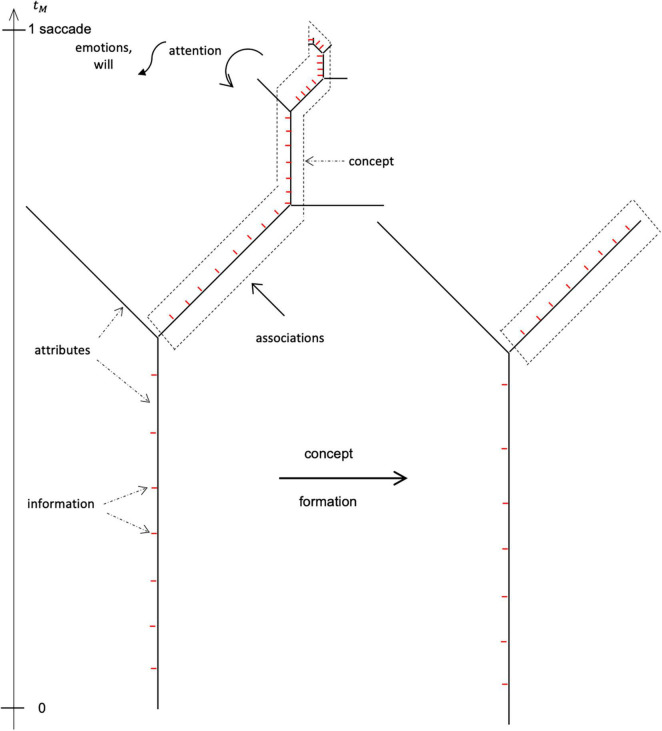
Mechanics of information, concept, association (memory), attention, and volition in consciousness. Coordinate axes (attribute axes) are the stuff of *consciousness* (correlate of physical space) in the Mental Maxwell model. The maximum mental velocity *c*_*M*_ = 7 ± 2 attributes/saccade is finite; therefore, only ∼7 attribute axes are laid down in consciousness within a mental time *t*_*M*_ = 1 saccade (vertical axis). In this representation, the axes are not Euclidean, but connected in a tree. Some axes populate with information (correlate of energy; red) at the same rate, *c_M_*. A number of the populated axes amalgamate to begin forming a concept (correlate of a physical particle; dashed lines). The concept can exchange associations (momentum correlate) with other concepts in memory. Once a concept is formed, its axes can collapse into a single attribute. This prunes the consciousness tree effecting a decrease in information and in consciousness. Attention (force correlate; curved arrow) is the curvature of consciousness-time and can accelerate, decelerate, or divert concept formation. Will and the emotions (correlates of physical yank; wiggly arrow) accelerate attention itself. Since behavior is organized on the concept level, there are thus multiple inputs regulating transitions between concepts and consciousness in a moment-to-moment mental scene.

Figure 2 illustrates the mental mechanics of concepts and consciousness in MM. One can model consciousness as an *N*-dimensional Euclidean space, but there are alternatives. One is a fractal space, such as a Cayley Tree (O’Neill and Schoth, under review). This is similar to Khrennikov’s (2000) p-adic trees of ideas. Staring from a trunk, the tree splits into limbs, branches, and sub-branches. Each branch is a coordinate axis, an attribute, unto itself. The overall tree is the scene, the coordinate system. The quantity of consciousness is the total number of branches manifest at any moment. Each branch is populated with bits of information, from 0 up to a maximum. This information comes both from the sensorium and from memory (it can also exit to the motorium). Certain attributes in certain branches in certain contexts trigger associations, the MM correlate of linear momentum (see “Mental Learning, Habit, Association ⇔ Physical Linear Momentum”), to flow in from memory (note: flow into consciousness is algebraically positive in MM). This supports concept formation. Alternatively, other attributes in other patterns in other contexts produce novel associations that are *learned* (associations going into memory are algebraically negative). Note that learning and recognition take place on the concept, not the attribute, level, occasioning less demand on memory. A concept in MM is an amalgam of multiple axes (for simplicity, we assume them contiguous) each bearing a load of information. When a concept is formed, the axes fuse leaving a stump. That implies a *drop* in consciousness when a concept forms, one sacrifices detail to conceive of the entity as a whole; when a concept dissolves, in contrast, when one sees the trees rather than the forest, detail and consciousness increase.

Mental velocity is finite – it takes time to erect (or tear down) conscious axes. If one concept is being constructed at the maximum rate, *c_M_*, then $\left(7\frac{\text{attribute}}{\text{saccade}}\right)\left(1\text{saccade}\right)=7\text{attribute}$ are laid down in 1 saccade. If each attribute triggers one association (1 link) and is again populated with information at *c_M_*, we have $\left(1\frac{\text{link}}{\text{attribute}}\right)\left(7\frac{\text{bit}}{\text{link}}\right)=7\frac{\text{bit}}{\text{attribute}}$. The information of the concept $\left(7\frac{\text{bit}}{\text{attribute}}\right)7\text{attribute}=49\mathrm{bit}$ is then numerically equal to $c_{M}^{2}$ and the mental inertia is $m_{M}=E_{M}/c_{M}^{2}=1\mathrm{bit}\cdot {\text{saccade}}^{2}/{\text{attribute}}^{2}$. Hence, mental inertia generally is the fraction of the maximum information that can be invested into (or extracted from) a concept within 1 saccade.

The tree-like coordinate system allows attributes to arise and to interconvert arbitrarily, as seen in the actual workings of the mind. For example, one moment we are looking at a leaf with its attributes of green, yellow, venation, roundness, texture, etc. The next moment we zoom out to take in the bough of which the leaf is a part. We then lose consciousness of the leaf attributes and instead see the blob of the bough as a concept with its own attributes. One attribute is “green,” another might be called “leafiness.” That is, the multiple leaf attributes have collapsed into a single bit; it now counts as “1 leaf” and is counted into the leafiness attribute of the bough. Through concept formation, some information is lost and some consciousness degenerates into information.

Figure 2 further indicates that attention (MM analog of force; see “Mental Attention ⇔ Physical Force”) is curvature of consciousness-time, acceleration – change in rate or direction – of the growth of consciousness about a concept. Will or emotions (MM correlates of physical yank; see “Mental Will, Emotion, Pain, Drives ⇔ Physical Yank”) are changes in rate or direction of attention itself. This matches the Schwartz et al. (2005) idea of will as “attention density,” by which they meant attention per unit *time*. It is also James’s (1890) conclusion, “Will is effort of attention.”

#### Mental Learning, Habit, Association ⇔ Physical Linear Momentum

Linear momentum in physics is the time integral:


(3) p=∫Fdt=-∫∇⁡Udt

of force or of the negative potential energy gradient. The MM correlate of momentum is *learning*, building *habits* or *associations* between concepts. We use these interchangeably in MM, preferring the term “association.” The bonds between attributes forming concepts and between concepts in scenes change or persist dynamically. That is, there is a reactive (time-dependent) character to the emergence into and disappearance from consciousness of concepts, the mental inertia. As mentioned, it takes time to erect attribute axes and to fill them with information. On the other hand, when an association links two concepts, appearance of one in consciousness tends to drag the other after it. MM accounts for this by appending association axes to the attribute axes, making a phase space of consciousness analogous to the position-momentum phase space in physics.

Physical potential energy is analogous in MM to the information linking (or disjoining) concepts in memory. The attribute space of memory has a gradient like that of Eq. (3). Thus, the architecture of memory, the steepness or shallowness of the gradients between concepts, propels them into consciousness. Concept boundaries in memory are marked by high information gradients, conceivably deriving from the gradients in consciousness that separate concepts from backgrounds. Between a hanging leaf and surrounding sky, for example, greenness drops off suddenly and blueness rises sharply, two steep gradients in attribute space. Such may be carried over into memory, then perhaps further sharpened through repeated exposures to similar leaves over time, as the concept is learned. The concept becomes more strongly bonded to certain fellow concepts and more clearly differentiated from others. Different concepts linked by associations share information gradient boundaries along one or more dimensions and, hence, tend to be propelled successively into consciousness. In mechanics, a body of given mass has greater or lesser momentum according to its velocity; in the mind, a concept of given mental inertia has greater or lesser tendency, according to the strength of its associations, to transition between memory and consciousness.

Perceptual associations bundle attributes into concepts (objects, events, words,…) Concepts then bundle into scenes (and into tasks within scenes; see “Behavioral Task ⇔ Thermodynamic System; Task-Relevant and -Irrelevant Meaning ⇔ Thermodynamic Work and Heat”). The scenes warp from moment to moment as the coordinate origin translates along a timeline. Perception defines object boundaries, in part through learned associations. The spatial coordinates of the phase space of consciousness are pure attributes; its momentum coordinates are tendencies for attributes to fuse into concepts, the associations.

The MM unit for association is 1link = 1 bit ⋅ saccade/attribute. Habit is an RDoC construct (Morris and Cuthbert, 2012), as is (associative) Declarative Memory. Habit acquisition is thought to occur *via* circuits entailing premotor or other prefrontal cortex, striatum, globus pallidus, thalamus, and subthalamic nucleus (Graybiel, 1995). Declarative memory is supported at least partly by the hippocampus and other mesial temporal structures (Squire, 1992; Cohen et al., 1997).

#### Mental Attention ⇔ Physical Force

*Force* is an indefinable of physics – in simplest form a push or pull. The MM correlate of force is *attention*, which pushes or pulls concepts into consciousness. This choice is intuitive; it is mentally effortful to attend to a neutral or unpleasant concept, or to disattend a delightful or worrisome concept. This effort feels subjectively like muscular force exertion. We are mentally drawn toward or away from concepts according to their emotional valence in a given context, and we counter these emotional drives with willful effort. Integrated over distance, force yields energy; integrated over time, it yields momentum. Hence, force is the gradient (spatial derivative) of energy and the time derivative of momentum. Just as force is required to change the velocity of a mass, in MM attention is required to shift concepts in and out of consciousness. When we attend to a concept, information from that concept streams into consciousness, and into memory. Stare, for example, at a plastic water bottle before you on a table. First you see the bottle as a whole, but now focus on the cap, expending a slight effort to make and hold the shift. Information streams in preferentially about the cap. You notice rills in its sides, a nick in one rill, a bubble under the surface, a skew in its bearing on the threads, etc. The rest of the bottle does not disappear as one focuses on these progressively finer features, but it does fade in detail. Less information comes from the attributes of the body of the bottle, more from the attributes of the cap. Similarly, one pays greater motor attention to a manipulated object being focused on than one not focused on, and one outputs more information into the attributes of the focused-upon object. A whittler, for example, subtly re-angles the cutting blade, carefully presses with the thumb, twists the stick by degrees, tilts gradually. Thus, new attributes emerge as attention is paid; attributes disappear when attention departs and the flow of information shifts from the attributes of one concept to those of another. This is quite like the acceleratory action of physical force.

Attention causes information to stream into (or out of) memory. In MM, mental inertia is the strength of the association between attributes to form concepts or between concepts to form scenes in memory. Attention paid in MM to attributes bundled into a concept or to concepts co-occurring in context over time (especially repeatedly) builds and strengthens these associations. This is learning. Thus, attention in MM is both information per consciousness channel (attribute) and learning per unit time.

Thus, we get the **Learning-Attention Relation**


(4) $$\begin{array}{c} {\text{p}}_{M}=\int {\text{F}}_{M}dt_{M}\;\text{or}\\ \{\text{learning}\}=\int \{\text{attention}\}d\{\text{time}\} \end{array}$$

The intuitively appealing analogy between attention and force leads to a picture of attention as information gradient in mental attribute space and as association-building per unit time. This resembles our concept of willpower as rate of change of attention (see “Mental Will, Emotion, Pain, Drives ⇔ Physical Yank”).

The MM unit of attention is 1 bit/attribute=1link/saccade, i.e., attention is both information per conscious channel and association per unit time. The attention construct is an RDoC domain (Morris and Cuthbert, 2012). Frontal and parietal cortices and thalamus are major brain structures thought to underlie attention (Coull, 1998).

#### Equation for Consciousness

In MM there is a **Dual Function of Attention**:


(5) $$\begin{array}{c} F_{M}∼\frac{E_{M}}{l_{M}}∼\frac{p_{M}}{t_{M}}\;\left\{\text{attentiion}\right\}\\ \;∼\frac{\left\{\text{information}\right\}}{\left\{\text{consciousness}\right\}}∼\frac{\left\{\text{association}\right\}}{\left\{\text{time}\right\}}. \end{array}$$

Rearranging we obtain an **Equation for Consciousness**


(6) $$\begin{array}{c} l_{M}∼\frac{E_{M}}{p_{M}/t_{M}}\;\left\{\text{consciousness}\right\}\\ \;∼\frac{\left\{\text{information}\right\}}{\left\{\text{association}\right\}/\left\{\text{time}\right\}}. \end{array}$$

Thus, unexpectedly, consciousness in MM is information per rate of association or per *coincidence rate*. For an association is a registration of a coincidence, a content in a context or two concepts co-occurring within a time interval. This is an example of an unexpected finding of the kind we hope the MMR will produce eventually for the field.

Compare Eq. (6) to everyday consciousness. Eq. (6) reasonably implies that, all things equal, the more information, the more consciousness. For example, routinely accepting a quarter in change at a newsstand, you notice little about the coin. You reduce it to its symbolic, functional value: “25¢ toward what I’m owed.” Mentally, it manifests as a quick flash in the palm. That is *less* information. However, if you are conscious of the quarter, you notice its elevated rim, its raised portrait and flat background, its lettering fonts, light and shadow, heft, etc. That is *more* information. Thus, the more information, the more consciousness is intuitive. Next, Eq. (6) implies, all things equal, the more *time*, the more consciousness. Clearly, focusing on an object longer, we do grow more conscious of it. However, when we shift gaze rapidly without concentrating on any one thing, we are less conscious. Finally, associations are in the denominator of Eq. (6). The more associations made, i.e., the more bundles of attributes that have been fused into concepts, like the quarter, perhaps a nickel and dime in change, the newspaper under your armpit, the brim of your hat, etc., the less consciousness there is. Each concept formed fuses several attribute axes thereby reducing consciousness. To summarize, “You are more conscious when you absorb more information from (put more information into) one thing for longer than when you absorb less information from (put less information into) several things for a shorter time.” A Zen monk might agree.

Equation (6) might serve as an operational definition of consciousness (Greenfield and Collins, 2005), possibly ultimately relatable to the “assemblage” unit of measure for consciousness suggested by these investigators to quantify the space–time dynamics of cell assemblies. This formula might seem a purely physical definition, yet it still does not fully close the explanatory gap (Levine, 1983) between physical properties and mental sensation. It provides a quantitative measure for the magnitude of consciousness as the number of axes. This number might relate to physical quantities, for example certain positron emission tomography (PET), fMRI, and magnetic resonance spectroscopy (MRS) measures of consciousness (Shulman, 2001; Shulman et al., 2003, 2009) – provided that the contribution of arousal (see “Mental Arousal ⇔ Thermodynamic Temperature”) can somehow be removed from these neuroimaging endpoints. However, the ordering of the attribute axes (e.g., the indexing of the axes, the branch hierarchy of the Cayley Tree of Figure 2) that constitutes the “mind code” between meaning and information remains in the mental realm. As a further aspect, Eq. (6) is consistent with panpsychism (Strawson, 2006; Tononi and Koch, 2015). If you stare into the eyes of a cat, more so with monkeys, apes, and cetaceans – even resisting sentimentality and anthropomorphism – there is a strong sense that there is “someone in there, staring back at you.” These animals respond to their environment, sometimes even to objects recently placed out of perception, in ways that suggest they possess a kind of mental imagery similar to that of humans. People who work with such animals are occasionally surprised by their apparent intelligence and ingenuity, particularly when motivated. Panpsychism is the idea (with many distinguished present-day and historical advocates) that consciousness inheres not only in human beings, but – presumably to a lesser degree – also in animals, or even inanimate objects. Consciousness is a ubiquitous property of nature in panpsychism. This is consistent with the MM notion of consciousness as a fifth indefinable of physics (see “Mental Consciousness ⇔ Physical Volume”). Further, a common complaint against panpsychism is that it has little to say beyond its proposed unity of mind and matter. Eq. (6) is compatible with panpsychism in that it implies that any entity – perhaps even certain kinds of machines – that is capable of performing operations in bundles of information per association rate is potentially conscious. This might open an avenue for panpsychism to be more productive in that it posits a metric for evaluating how conscious various entities are and which entities are or are not conscious. One issue thereby, however, is to what extent the factor “association” in Eq. (6) applies to mental (e.g., mnemonic) vs. merely statistical associations.

#### Mental Will, Emotion, Pain, Drives ⇔ Physical Yank

In physics, yank is mass times jerk. It is the first time derivative of force, the second time derivative of momentum. Elsewhere (O’Neill et al., 2019), we presented the mental correlates of yank – will (volition), the emotions, pain, and drives. Briefly, in MM, will is the capacity to execute thoughts and motor actions counter to emotions, habit, and/or external resistance. Will and emotions have a similar character but often act in opposition. Both are time rates of change of attention, i.e., they produce, destroy, or redirect attention. This is again James (1890), “Will is effort of attention.” Scenarios in physics with non-zero yank are often unstable with high energy expenditure. Similarly, in behavior, willful efforts are prone to sudden collapse and can be energetically costly. Like jerky trajectories in physics, willful behavior has a chunkier time scale with likely sudden turns, breaks, and course reversals.

The MM units of will, emotions, etc., are 1 bit/(attribute ⋅ saccade) = 1 link/saccade^2^. The will appears in RDoC as Cognitive (effortful) control; the emotions fall under Negative and Positive valence systems. One view of will, pain, and emotions associates them with the cingulate cortex. Middleton (2009) charted brain locations where neuroimaging or other physiological responses were associated with the urge to move a body part, incited by fear, pain, sadness,…; exercise of free will; or symptoms of obsessive-compulsive disorder, a disease intimately related to will and emotion (O’Neill and Schwartz, 2005). Most sites for all causes were in cingulate subregions, implying that fear, pain, emotions, and will all have at least one cingulate functional center. The various subregions drive responses (skeletomotor, visceromotor, glandular,…) of the organism to behavioral scenarios in association with subjectively experienced (and externally expressed) emotions. There is a good deal of evidence linking cingulate subregions to pain and emotions (Vogt, 2005, 2019). In concert, these emotions determine *approach-avoidance* behavior. Does a salesman avoid making a call due to fear? Does a weightlifter avoid pressing a barbell due to pain? Does an addict approach a crack pipe for pleasure (or to relieve craving)? Or does will overcome these drives? Hence, there may exist a functional anatomic basis for a common character for will, pain, and emotions like fear, sadness, happiness, etc.

### Mental Familiarity-Novelty ⇔ Electric Charge

*Charge* is a core indefinable of physics. Charges are sources of electrostatic attractive and repulsive forces acting on other charged bodies to move them across space. The MM correlate of charge is the *familiarity* (positive charge) or *novelty* (negative charge) of a concept. We discuss charge only briefly as it is not in the MMR.

Just as charges exert attractive and repulsive forces on other charged bodies, the familiarity or novelty of a concept with respect to other concepts in a mental scene is a source of attention that pulls or pushes it into or out of consciousness. We examine four cases. The first is *familiar–familiar* corresponding to positive–positive charge interactions. Like mutually repellant positive charges, multiple familiar objects in a scene evade attention, are pushed out of consciousness. For example, several familiar pieces of furniture decorating a room fade into the background if one does not actively attend to them or no unexpected event draws attention to them. The second is *novel–novel* (negative–negative). Like mutually repellant negative charges, too many novel objects in a scene compete for attention, pushing each other out of consciousness. Examples include relics in an antic shop, knick-knacks at a carnival, or family photographs on an acquaintance’s mantle. It requires effort of attention to focus on any one item in the crowded field. Third is *familiar–novel* (positive–negative). Like the attractive force on a negative charge surrounded by positive charges, a novel item amidst many familiar objects calls attention, is pulled into consciousness. For example, a single green parrot in a flock of black crows or an unknown player’s name on a sports team’s roster draws attention. Finally, *novel–familiar* (negative–positive). Like the attractive force on a positive charge surrounded by negative charges, a familiar object amidst many novel objects calls attention, is pulled into consciousness. For example, a childhood friend attracts more attention unexpectedly encountered overseas than seen back home among old buddies at the local tavern.

In each case, attention pushes the object into or out of consciousness. Clearly, familiarity and novelty are matters of memory. Physical particles form electrochemical bonds to aggregate into atoms, molecules, etc. Analogously, associational bonds of memory are strengthened by the repeated appearance of a concept in a particular context or by the exceptional peculiar appearance of a concept in an unusual context. The mnestic forces in these bonds may be similar to attention pushing concepts into and out of consciousness, recalling the spin-glass model (Hopfield, 1982).

The unit of familiarity–novelty is 1 *distinction*. Although not in RDoC, familiarity (Aggleton and Brown, 2006) and novelty (Antunes and Biala, 2012) are long-standing topics in neuroscience. The amygdala may be one brain site for novelty/familiarity detection (Halgren et al., 1980, 1994; Murray et al., 2014).

## Mental Analogs of Thermodynamic Variables

### Mental Salience ⇔ Physical Stress

*Pressure* or *stress* in physics is force per unit area. The MM correlate of these is *salience* or attention per consciousness background, i.e., attention paid to one channel in consciousness divided by all other channels. Thus, attention surface density is the amount of information coming in (or going out) relative to the size of the background, the salience of the attribute attended to. If, for example, a palm tree is high compared to its width, it is salient for height and attention is drawn to look up at it. It requires effort of attention to note less salient aspects of the palm like its knobby base or rough bark. Intuitively, it is clear that an object stands out (is salient) against a sparse, homogeneous background (fewer attributes) and that an object is less salient against a busy, complex background with many attributes. A palm tree is easier to see in the desert than in the jungle. Note that salience also increases (or decreases) by raising or lowering attention independent of background.

The MM unit of salience is 1 bit/attribute^*N*^. Salience is again not in RDoC but is widely accepted and long-investigated in cognitive psychology (Santangelo, 2015). The “salience network” (Seeley et al., 2007) is a canonical resting-state fMRI network including the anterior middle cingulate, orbitofrontal cortex, and insula.

### Behavioral Task ⇔ Thermodynamic System; Task-Relevant and -Irrelevant Meaning ⇔ Thermodynamic Work and Heat

The thermodynamic distinction between heat and work depends on system boundaries, on the level at which the observer arbitrarily tracks the system in detail. Work moves system boundaries; heat (for closed systems) does not. The MM analog of a thermodynamic system is the *task*: the attempt to complete an *action*, *thought*, or *sentence*. The concepts employed in the task take on various *syntactic roles*, e.g., agent, direct object, and indirect object. We use “syntax” here generally, encompassing not only sentence construction but motor actions, etc. For example, if you hurl a rubber ball against a brick wall, your hand is the agent, the ball the direct object, the wall the indirect object, to hurl the verb, rubber and brick adjectives, etc., even if you never describe your actions in a sentence. Just as in different natural languages or constructions within the same language, subject, object, etc., can be shifted, so, too, can the mind variously construe the roles in a motor action. For instance, are you running your finger along the doorframe or is the doorframe guiding your finger? Are you sitting on the sofa or is the sofa supporting you? Our mental analog for work is *task-relevant meaning*; our analog for heat is *task-irrelevant meaning*. That means information from concepts pertinent to the task, respectively, information from concepts not pertinent to the task. For example, while throwing the ball, seeing that the sky above is gray, hearing a car horn honk in the distance, recalling that you are late on rent,… are all irrelevant to the task, while the heft of the ball, its elasticity, distance to and height of the wall, etc., are all relevant.

Task-relevant and task-irrelevant meanings have the same unit as information, 1 bit. “Task” as a concept has been a mainstay of cognitive psychology for decades. Different tasks have different centers and networks as brain bases. The foregoing is evocative of the notion that the brain is organized around meaning rather than information (Freeman, 1975; Pribram, 2013).

#### Meaning Is Information in Transit to Behavior

Consciousness erects a set of coordinates each representing a different attribute at each instant. Information is the amount of each attribute. We distinguish information from *sense* or *meaning*. In physics, the coordinates (−1 m, 11.5 m, 3.2 m) constitute information. If the observer faces one way, the triple means a point 1 m left, 11.5 m forward, and 3.2 m up, but turning around, the same coordinates mean 1 m right, 11.5 m behind, and 3.2 m up. If a coordinate system is oriented one way, the information indicates one point and if it is oriented another way, the identical information indicates an entirely different point. Wittgenstein (1958) provides simpler examples. Which of the two pairs of arrows = > = > and = > < = point in the *same* direction? Placing a marker thus = > = >|, = > < =| shows it is the pair on the left (both point rightward), but placing the marker thus = >| = >, = >|< = shows it is the pair on the right (both point inward). The answer, even in simplest cases, depends on convention, i.e., the coordinate system. Information alone has no sense. In the same way, information in the mental world has no meaning without *context*. Imagine, for example, a half-full cup of coffee on a table. In the context “beginning breakfast,” the cup means warmth, pleasure, nourishment,…, but in the context “cleaning up,” the same cup (at identical temperature, etc.) means garbage, labor, disgust,… Meaning depends on context. In thermodynamics, heat and work are forms of energy in transit. A gas pushing on a piston transfers energy (work) into it; a torch warming the cylinder enclosing the gas transfers energy (heat) into it,… In MM, meaning is *information in transit* to behavior. Information from the coffee cup heading into the behavior “eating breakfast” means “nourishment”; information from the cup heading into “cleaning-up” means “garbage.”

A mental eigenvector basis as described above lends meaning because it is the set of possible messages (Shannon, 1948) out of which the message produced is selected. That is, the message produced is a linear combination of eigenvectors in the basis. Shannon: “If the sending device is equally likely to send any one of a set of *N* messages, then the preferred measure of ‘the information produced when one message is chosen from the set’ is the base two logarithm of *N* (this measure is called self-information).” Attention does the *choosing* and thereby produces meaning.

Our concept of meaning as information in transit to behavior resembles Gibson’s (1979) “affordance” (Neisser, 1997). For example, in an office, the floor *affords* walking; the doors *afford* passage, etc., “what this means is simply that I could walk across the room and go out the door (if I wanted to). Seeing a glass of water at hand I could drink from it, or for that matter throw it across the room. Affordances can often be directly perceived: I can *see* that the floor affords walking, the glass drinking. To be sure, I don’t see everything. Every situation objectively offers infinitely many affordances for any given individual, of which only a few are perceived and even fewer realized in action.” At each moment, one is surrounded by and can imagine more concepts (objects, sounds,…) each loaded with information and each acquiring meaning as soon as one envisions them being employed in behavior. Meaning is inherently subjective; it concerns conscious experience. Moreover, it is always translatable into terms of the *behavior* of an organism emerging from its history, physical and mental limitations, responses to stresses, and efforts to fulfill needs.

Meaning comes into RDoC under Language Behavior and is widely employed outside RDoC (Pulvermüller, 2013). Neuroimaging and lesion studies find brain bases of semantic meaning across the cerebral cortex (Pulvermüller, 2013). This is affirmed by fMRI studies of meaning in narrative context (Huth et al., 2012), which localize word meanings to anterior visual, mesial and lateral parietal, auditory, and lateral prefrontal cortices.

### Mental Arousal ⇔ Thermodynamic Temperature

The *temperature* of a thermodynamic system indicates the variance or breadth of distribution of energy across the available energy modes. For cold systems (of particles), the energy is concentrated in a few low-energy modes inhabited by most particles; for hot systems, the energy is more broadly distributed across modes so there exist more particles with higher energies.

The MM correlate of temperature is *arousal*. Arousal in MM parameterizes the distribution of information across concepts. At low arousal, it is hard to focus on more than one thing at a time, most information comes from (and goes to) one or a few sources (and sinks). In high arousal, information input and output are spread across multiple sources and sinks. Higher-level information transfer can occur for a number of concepts. Suppose, for example, in lethargy – low arousal – you stare vacantly at a bottle before you and fumble to grasp it, unaware of much else. Most information comes from and goes to the bottle (a few modes). The information is low-grade, a dull awareness of label and shape and gross digital movements. In a hyperaroused state, in contrast, you may chat incessantly, clear all bottles from the table, wipe it down, wash the dishes, and take out the trash. You input and output finely detailed information from and to numerous concepts. Similar relationships apply during weariness, agitation, etc.

Temperature indexes rather than directly measures internal energy. The actual width (in J/particle) of the energy distribution is *kT*/2 (with *k* the *Boltzmann constant* in J/K ⋅ particle), the magnitude of spontaneous energy fluctuations in the system at equilibrium. The higher above *kT*/2 a transition between energy levels is, the less probable a particle will undergo that transition spontaneously. Analogously, we choose an indirect index, “degrees GCS” (for the Glasgow Coma Scale; Teasdale and Jennett, 1974), as unit of arousal. We can also define a “mental Boltzmann constant,” *k_M_*, with units of bit/GCS ⋅ concept. *k*_*M*_*T*_*M*_/2 then has units of bit/concept and yields the variance of the information distribution across concepts. It also yields the size of the usual fluctuations of information between memory and consciousness at mental equilibrium (see “Mental Meditative State ⇔ Thermodynamic Equilibrium”). Arousal is in RDoC. Its brain bases include basal forebrain, brainstem cholinergic nuclei, locus coeruleus, and the midbrain reticular formation (Schiff, 2008).

### Mental Distraction ⇔ Thermodynamic Entropy

The MM correlate of *entropy* is *distraction*. Distraction is task-irrelevant information per unit arousal, just as entropy is heat per unit temperature. Its units are bit/GCS ⋅ concept. Distraction is only represented indirectly in RDoC, as inverse attention, but has long been an important neurocognitive endpoint (Sarter and Paolone, 2011; Greenwood and Parasuraman, 2016). Various brain sites are associated with distraction, including the mediodorsal thalamus (Sarter and Paolone, 2011), superior parietal cortex (Greenwood and Parasuraman, 2016), and anterior and posterior middle cingulate (Heckers et al., 2004).

### Mental Meditative State ⇔ Thermodynamic Equilibrium

The thermodynamic Maxwell Relations are written for quantities assumed to be functions of state at *equilibrium*. The MMR are likewise proposed to be most accurate at mental equilibrium. The MM correlate of equilibrium is the *meditative state*. A non-equilibrium physical state depends on history; an equilibrium state is independent of history. Similarly, in normal wakefulness, a brain continually records and recalls memories, while in meditation, one lets thoughts and sensations pass through consciousness without registration and without reacting to memories arising. Like equilibrium, meditation is dynamic: as each thought or sensation calls attention by evoking emotion, it is swiftly counterbalanced by an equal and opposite exertion of the will. For an isolated system, entropy is maximal at equilibrium, i.e., work is maximally dissipated into heat. In meditation, one pursues a single, simple task (classically, focusing on the breath). Task-relevant information (work correlate) is low. The great mass of other thoughts and sensations flowing through consciousness is irrelevant. Therefore, task-irrelevant information and task-irrelevant information per unit arousal – distraction – are maximal, just as entropy is maximal in thermodynamic equilibrium. Reddy and Roy (2018, 2019) analyzed the study of mediation in contemporary neuroscience, making useful recommendations, including for definitions of the meditative and baseline states.

## The Mental Maxwell Relations

Plugging the MMR analogs of the thermodynamic variables into the thermodynamic Maxwell Relations yields the **Mental Maxwell Relations**:


(7) $$\begin{array}{c} {\left(\frac{\partial T_{M}}{\partial \varepsilon_{M}V_{M}}\right)}_{S_{M}}=-{\left(\frac{\partial \sigma_{M}}{\partial S_{M}}\right)}_{\varepsilon_{M}V_{M}}{\left(\frac{\partial \left\{\text{arousal}\right\}}{\partial \left\{\mathrm{consc}\mathrm{deform}\right\}}\right)}_{\left\{\text{distraction}\right\}}\\ \;=-{\left(\frac{\partial \left\{\text{salience}\right\}}{\partial \left\{\text{distraction}\right\}}\right)}_{\left\{\mathrm{consc}\mathrm{deform}\right\}}\\ \;{\left(\frac{\partial T_{M}}{\partial \sigma_{M}}\right)}_{S_{M}}={\left(\frac{\partial \varepsilon_{M}V_{M}}{\partial S_{M}}\right)}_{\sigma_{M}}{\left(\frac{\partial \left\{\text{arousal}\right\}}{\partial \left\{\text{salience}\right\}}\right)}_{\left\{\text{distraction}\right\}}\\ \;={\left(\frac{\partial \left\{\mathrm{consc}\mathrm{deform}\right\}}{\partial \left\{\text{distraction}\right\}}\right)}_{\left\{\text{salience}\right\}}\\ {\left(\frac{\partial \varepsilon_{M}V_{M}}{\partial T_{M}}\right)}_{\sigma_{M}}=-{\left(\frac{\partial S_{M}}{\partial \sigma_{M}}\right)}_{T_{M}}{\left(\frac{\partial \left\{\mathrm{consc}\mathrm{deform}\right\}}{\partial \left\{\text{arousal}\right\}}\right)}_{\left\{\text{salience}\right\}}\\ \;=-{\left(\frac{\partial \left\{\text{distraction}\right\}}{\partial \left\{\text{salience}\right\}}\right)}_{\left\{\text{arousal}\right\}}\\ \;{\left(\frac{\partial \sigma_{M}}{\partial T_{M}}\right)}_{\varepsilon_{M}V_{M}}={\left(\frac{\partial S_{M}}{\partial \varepsilon_{M}V_{M}}\right)}_{T_{M}}{\left(\frac{\partial \left\{\text{salience}\right\}}{\partial \left\{\text{arousal}\right\}}\right)}_{\left\{\mathrm{consc}\mathrm{deform}\right\}}\\ \;=-{\left(\frac{\partial \left\{\text{distraction}\right\}}{\partial \left\{\mathrm{consc}\mathrm{deform}\right\}}\right)}_{\left\{\text{arousal}\right\}} \end{array}$$

where “consc deform” is “consciousness deformation” and subscripts for *n*_*iM*_ are omitted. The reciprocals also constitute MMR.

Like the thermodynamic Maxwell Relations, the MMR make predictions that are not obvious or trivial. For example, while it is reasonable to think that arousal increases with consciousness for constant distraction, it is not obvious that this should occur at the same rate that salience decreases with increasing distraction. It is reasonable to expect that arousal increases with salience for constant distraction, but less expected that consciousness increases with distraction for constant salience. It is expected that consciousness increases with arousal for constant salience and perhaps also that distraction decreases with increasing salience for constant arousal. Finally, it is reasonable that salience increases with arousal for constant consciousness, but less expected that distraction increases with increasing consciousness for constant arousal. All these predictions might be tested, ideally on meditating subjects.

### Ideas for Experimental Testing of the Mental Maxwell Relations

We anticipate that, with ingenuity, one can design cognitive experiments to test each MMR. Thereby, consciousness may be operationalized as the number of distinguishable sensory or motor attributes in a scene and may be manipulated by limiting such. Manipulations already used by experimenters include filtering out parts of the visual field or audio spectrum, or anesthetizing derms of the skin. Salience may be calculated as bits of information in a sensory or motor attribute channel attended to by the subject divided by the number of all attribute channels minus 1 (factorial). Arousal may be assessed, as mentioned, by instruments such as the GCS or by EEG, pupillometry, etc., proxies. Distraction may be quantified as bits of task-irrelevant information, introduced into or excluded from the experiment, normed to the subject’s arousal level. With suitable measurement and quantification, one might thus test the MMR experimentally. The MM mental variables and their thermodynamic correlates appear in Table 1.

**TABLE 1:** Analogy between thermodynamic and mental variables.

Symbol	Thermo	Units	Mental	Units
*t*	Time	s	Time	saccade
*l*	Length	m	1D consciousness	attribute
*A*	Area	m^2^	(N-1)D-consciousness	attribute*^N–1^*
*V*	Volume	m^3^	ND-consciousness	attribute*^N^*
ε	Strain	Unitless	Consciousness deformation	Unitless
**v**	Velocity	m/s	Mental velocity	attribute/saccade
*m*	Mass	kg	Mental inertia	bit ⋅ saccade^2^/attribute^2^
−	Particle	Particle	Concept	concept
−	System	−	Task	−
*i*	Chemical species	−	Syntactic role	−
μ_*i*_	Chemical potential	J/particle	Syntactic potential	bit/concept
**p**	Momentum	kg ⋅ m/s	Association, habit, learning	link
**F**	Force	N	Attention	bit/attribute = link/saccade
*P*	Pressure	Pa	Salience	bit/attribute*^N^*
σ	Stress	Pa	Salience	bit/attribute*^N^*
**Y**	Yank	N/s	Willpower, emotions, drive	bit/attribute ⋅ saccade = link/saccade^2^
*E*	Energy	J	Information	bit
*U*	Internal energy	J	Internal information	bit
*Q*	Heat	J	Task-irrelevant meaning	bit
*W*	Work	J	Task-relevant meaning	bit
*T*	Temperature	K, C°	Arousal	°GCS
*kT*/2	Boltzmann energy fluctuation	J/particle	Spontaneous information fluctuation	bit/concept
*S*	Entropy	J/K particle	Distraction	bit/°GCS ⋅ concept
*q*	Electric charge	C	Familiarity–novelty	distinction

*Time – the one attribute a mental scene (context, cycle) retains when all else is stripped away; number indexing distinguishing mental scenes.*

*Consciousness – the scene or coordinate system that gives sense or meaning to information; each axis is an attribute into or out of which information flows; 1D – a single axis or attribute in consciousness (N-1)D N-1 axes, ND – N axes; each axis is a degree-of-freedom.*

*Mental inertia – tendency of a concept to bundle attributes, tendency for attributes to evolve together as a concept; resistance to concept dissolution. The information in the associative bonds of a concept in memory can translate into mental inertia when the concept emerges into consciousness.*

*Concept – a bundle of attributes possessing information by virtue of its position in memory (“potential information”) and its mental inertia (“kinetic information”).*

*Attention – information per attribute flowing into or out of a concept (like force exerted on or by a particle), equal to learning, habit, or association per unit time (learning = time × attention).*

*Salience – attention per unit background.*

*Drive – attention generated or consumed per unit time, can be innate, as hunger, thirst, lust,… or can stem from learned emotional associations, e.g., reflexive aversions, ritual pursuits,… Can have long-enduring or explosive, spark-like character. Drives often push behavior away from the task at hand.*

*Will – like drive, attention generated or consumed per unit time. More often pushes behavior toward the task at hand.*

*Information – scaling of an attribute axis in consciousness, the smallest distinguishable increments along an attribute axis in bits (log_2_).*

*Internal information – information not manifest in consciousness, locked in memory bonds between concepts.*

*Meaning – information in transit to behavior; the context, axes in attribute space, set the meaning of a concept.*

*Task-relevant meaning – information that changes the attributes and/or mental inertia of those concepts playing a syntactic role in the task at hand.*

*Task-irrelevant meaning – information that changes the attributes and/or mental inertia of concepts other than those playing a role in the task at hand.*

*Task – completion of, or attempt to complete, an action, thought, or sentence; one pass through the Kant Cycle. A set of concepts each having, for the course of the Cycle, one or more syntactic roles.*

*Syntactic role – a particular configuration of information held by a concept by virtue of its attributes and mental inertia during a task in a given context; meaning can arise as the various concepts assume their syntactic roles during the execution of a task, analogous to the way heat (and work as pressure waves, etc.) can be released by the various chemical species interacting in a chemical reaction.*

*Arousal – index of variance of distribution of information across the concepts in consciousness; measured in °GCS (degrees Glasgow Coma Scale).*

*Spontaneous information fluctuation – the variance of the distribution.*

*Distraction – task-irrelevant information per unit arousal.*

*Familiarity-novelty – memory-based distinction between a concept and its context; can serve as attention source or sink, the way electric charge generates Coulombic forces of attraction and repulsion.*

In particular, for evaluation of consciousness one could project a complex visual scene, e.g., a landscape, to a proband (see Supplementary Material). Shortly afterward, one could decompose the scene into attributes and project images of the attributes in isolation (alternating with other attributes that were not present) and ask the proband to acknowledge with a clicker whether or not the attribute was in the picture. Such could be, e.g., colors without form, curvatures without other form or color (beyond neutral gray), and textures without form or color. The same could be done presenting audio mixtures of tones and timbres through headphones or mixtures of vibrations and other tactile qualities through pads affixed to derms, etc. Perhaps better, one could present a scene and subtly tune out (or tune in) individual attributes while the proband is watching. The proband is instructed to click when “something in the scene changes.” There are already a host of further candidate techniques in the literature for measuring many of the mental constructs here discussed, including physiological or neuroimaging proxies for some.

## Mental Kant Cycle ⇔ Thermodynamic Otto Cycle

**FIGURE 3:**
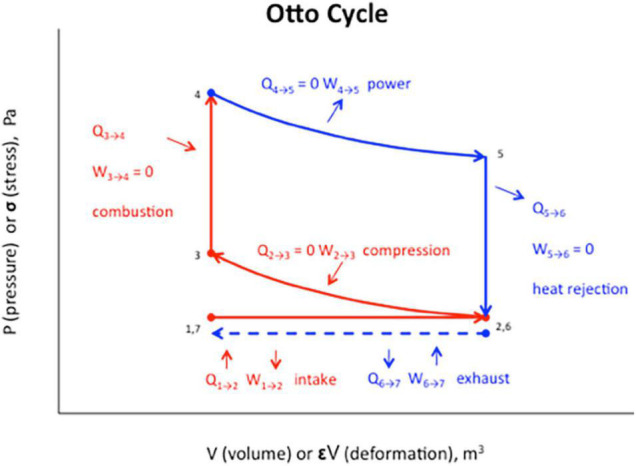
The Otto Cycle is the idealized basis of most spark-ignition gasoline engines. (Intake stroke, 1→2) a camshaft opens an intake valve allowing air from the atmosphere and fuel from the reservoir to enter the cylinder. This entering material brings with it the heat *Q*_1→2_ and expands system volume by pushing the piston, performing the work *W*_1→2_. (Compression, 2→3) the piston performs work *W*_2→3_ on the system by compressing it along an adiabat (*Q*_2→3_ = 0). (Combustion, 3→4) the spark plug ignites the fuel/air mixture releasing great heat *Q*_3→4_ into the gas. Since the heat is generated more quickly than the piston can follow, no work is done during combustion (*W*_2→4_ = 0). (Power 4→5) at high temperature, a good deal of work *W*_4→5_ is done as the gas expands, pushing back the piston. Ideally, there is no heat loss (*Q*_4→5_ = 0). During heat rejection (5→6), heat *Q*_5→6_ is lost as the temperature drops, but no work is done (*W*_5→6_ = 0). On the final stroke (exhaust, 6→7), the piston does work *W*_6→7_ on the system, expelling the products of combustion and any unburned air and fuel, along with heat *Q*_7→7_.

Investigators (e.g., Déli and Kisvárday, 2020; Deli, 2021; Deli et al., 2021) have recently employed thermodynamic cycles to study dynamics of higher brain functions intensively. One behavioral paradigm for moment-to-moment human mental processes is exemplified by a four-sentence sequence – “I see a tiger… I think I am in danger… I feel afraid… I run.” These represent the mental stages: perception, cognition, emotion, behavior. We call this the “Kant Cycle” as this paradigm has been attributed to Kant, who analyzed several of the relevant faculties of mind (Kant, 1781, 1788, 1790). We chose this paradigm as it is used in cognitive training (Peltier, 2009) and has high intuitive appeal. It provides several insights: perceptions are organized into concepts, including agents; cognition interprets concepts by their behavioral significance; emotions are fed by thoughts and drive actions; and actions initiate the next round of perceptions. Each stage is relevant to mental health: running through the Cycle quickly and automatically reinforces both good and bad habits, making them hard to break; false perceptions (as in hallucinations) lead to misconceptions, unexamined perceptions lead to stereotyped conceptions; negative thoughts (as in ruminations) foster negative mood states; unchecked emotions (as in impulses) lead to rash behavior. The stages also offer points of intervention: one can reexamine perceptions, one can reinterpret thoughts (cognitive restructuring), one can still thoughts (meditation), one can sit with an emotion rather than act upon it (distress tolerance); one can act opposite the direction the emotion is pulling (exposure and response prevention); and so forth. Note that many investigators reject this paradigm, citing evidence for alternatives. One can configure other cycles within MM corresponding to these alternative paradigms. One could, for example, construct a LeDoux Cycle in which emotion actually precedes cognition (LeDoux, 2015). Or for the famous James–Lange Theory, in which arousal instigates emotion (Dewey, 1894), one could construct a Dewey Cycle. However, the Kant Cycle, in any case, serves as a good heuristic since it is analogous to the Otto Cycle (Figure 3) of internal combustion engines. Perception, cognition, emotion, and behavior correspond thereby to the intake, compression, power, and heat-rejection strokes of the Otto Cycle; we add “drive-will” and “closure” as additional stages analogous to combustion and exhaust. Work done by or on the system is analogous to task-relevant information entering or leaving consciousness; heat absorbed by or released from the system is analogous to task-irrelevant information entering or leaving consciousness. The Kant Cycle deviates from the Otto Cycle in that there are no vertical (zero-work) or adiabatic (zero-heat exchange) legs; each process involves both task-relevant and task-irrelevant information. As mentioned, a mental system is the set of conscious and unconscious concepts active in the mind when it performs a task, e.g., formulates a sentence, executes a motor sequence.

**FIGURE 4:**
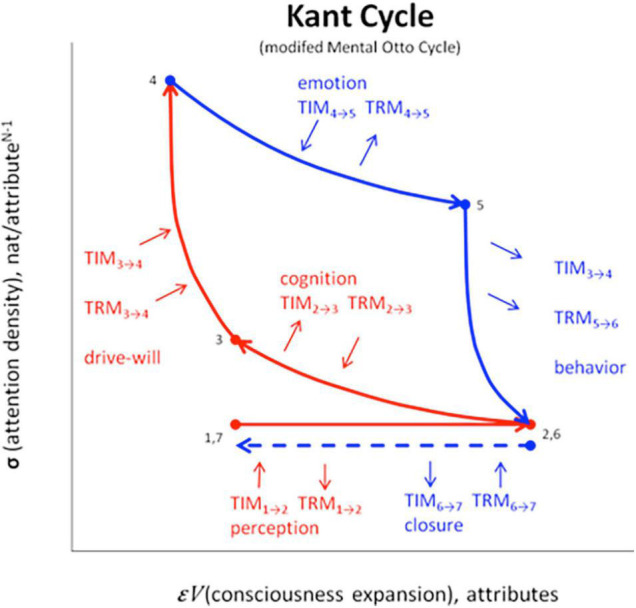
The Kant Cycle is a mental analog of the Otto Cycle. During perception (stroke 1→2), task-relevant meaning TRM_1→2_ flows from the mind into the sensory effectors (e.g., eye muscles), expanding consciousness at a low level of attention as task-irrelevant meaning TIM_1→2_ flows in from the sensors. During cognition (1→2), task-relevant and task-irrelevant meaning (TRM_1→2_, TIM_1→2_) enter the mind from memory dropping consciousness and raising attention. On leg 1→2, drives such as hunger, thirst,… and the will trigger a stream of task-relevant and task-irrelevant meanings (TRM_2→4_, TIM_2→4_) released from associations in memory into the mind. Attention and arousal rise sharply, although consciousness remains relatively fixed about the concepts recognized during cognition (3→4). During emotion (4→5), task-relevant and task-irrelevant meanings stream out of the mind into memory storage to form new associative bonds. Attention and arousal drop gradually as consciousness expands. During behavior (5→6), task-relevant and task-irrelevant meanings are transferred into the effectors (muscles and glands) as attention and arousal drop by modest expansion of consciousness. On the final stroke (closure 6→7), task-relevant information TRM_6→7_ enters from the motor sensors as consciousness compresses and task-irrelevant information passes to the sensory effectors to complete the Cycle.

Figure 4 illustrates the Kant Cycle. Resembling the PV diagram of the Otto Cycle, it plots salience as a function of consciousness for each leg of the cycle. On each leg, task-relevant and/or task-irrelevant meanings (information in transit to behavior) are taken in or given off by the system. The Cycle has the following strokes (analogous Otto strokes):

*1→2 Perception* (intake)
Task-relevant meaning enters sensory effectors (e.g., eye muscles). The effectors position the sensors to allow task-relevant (and task-irrelevant) meaning to flow in, expanding consciousness – analogous to the camshaft opening the intake valve to the fuel reservoir and/or atmosphere.

*2→3 Cognition* (compression)
Task-relevant and task-irrelevant meanings stream into consciousness from memory recall (analogous to the crankshaft in compression), drawing attention (pressure) and raising arousal (temperature). During cognition, influx of task-*relevant* meaning predominates.

*3→4 Drive-will* (combustion)
Triggered by prevailing drives (hunger, thirst, fatigue,…) and the will, task-relevant and task-irrelevant meanings again stream in from memory. However, on this leg, task-*irrelevant* meaning predominates. This leg is analogous to combustion, because, as spark plugs are force generators, rapidly unleashing energy from chemical bonds, so, too, do the will and other drives rapidly unleash information from hitherto untapped memory associations. Recognition, occurring during leg 2→3, is experienced as relatively less effortful, since it precedes will power (3→4) in the Cycle. During leg 3→4, there is great heightening of attention and increase in arousal, but little change in consciousness, for it is a brief epoch of fixation. The curve is nearly vertical because most associations entering our minds are irrelevant to the task at hand. Task-relevant information (deviation from the vertical) is delivered into the mind mainly by the will and actually entails reduction in consciousness (screening out the unnecessary).

*4→5 Emotion* (power stroke)
Task-relevant and task-irrelevant meanings stream out of consciousness into memory (analogous to the crankshaft in compression), forming new associative bonds. Thus, memories are drenched in emotion. Consciousness again expands as attention and arousal gradually drop.

*5→6 Behavior* (heat-rejection)
Both task-relevant and task-irrelevant meanings stream out to the effectors (analogous to exhaust or atmosphere). Note that the behavioral response consists of both task-relevant and task-irrelevant meanings, for actions always mix reason and emotion. However, attention and arousal drop as task execution is completed.

*6→7 Closure* (exhaust stroke)
Task-relevant meaning from motor (or other effector) sensors (analogous to the camshaft at exhaust) compresses consciousness at a low level of attention. As arousal drops, task-irrelevant meaning is transferred to the sensory effectors and can transition into task-relevant information as the system returns to the starting point.

Thus, using MM constructs, the Kant Cycle realizes a detailed thermodynamic analogy of higher brain functions.

## Decision-Making

There are many points of contact between MM and decision-making. *Alternatives* in decision-theory correspond to MM *tasks* being formulated and executed. Multiple tasks may undergo simultaneous and competitive construction in consciousness, perhaps according to drift diffusion, race, or attractor models (Deco et al., 2013) of the decision theory. Decision-making in MM thus includes the above-discussed steps of consciousness construction (laying down attributes, populating them with information), fusion of attributes into concepts, and chaining of concepts into tasks by a syntactic role. Difficulty assembling sufficient information to overcome a syntactic potential barrier may impede task completion and thus decision-making. That is, failure to identify a target, to set a bound, to produce a coherent motor response, to act with dispatch, etc., is like failure to insert a concept into the appropriate syntactic category. Syntactic potential is the MM correlate of the chemical potential. The latter is linked to the Gibbs free energy, widely applied to neuroscience by Friston (2010). *Consciousness* construction resembles *evidence accumulation* in the decision theory, indexed by the centroparietal positivity (CPP) EEG evoked potential (EP) component on the human scalp (O’Connell et al., 2012). The lateralized readiness potential (LRP) EP component, which builds up in proportion to muscular movement coherence (Kelly and O’Connell, 2013), may be a correlate of action-concept generation in MM. Decision-making is affected by *multiple sensory components*, not all pertinent to the decision; this is similar to the distinction of *task-relevant vs. task-irrelevant information* in MM. *Past evidence* impacts decision-making (O’Connell and Kelly, 2021) and is embodied in the MM *association* construct. Associations affect concept formation and drag concepts and chains of concepts ready for execution into consciousness from memory. Such recalled concepts enhance and create conscious scenes imparting desire, anxiety, etc., and facilitate scenario prediction. Incipient concepts assume roles in the tasks being formulated. *Target selection* in the decision theory may be signaled by the N2pc EP component (Loughnane et al., 2016). In MM, target selection is the assignment of one concept in the conscious scene to a *specific role* (e.g., indirect object). Continual engagement improves decision-making and correlates with posterior α-EEG power (Hanslmayr et al., 2007). In MM, *attention* improves decision-making by accelerating information loading into attributes (evidence accumulation) and diverting it into prefavored channels. *Urgency* in decision-making correlates with μ-β-EEG power (Kelly et al., 2021). In MM, urgency is affected by *will and emotions* accelerating attention. *Arousal* also affects decision-making (Murphy et al., 2016) and is associated with θ-EEG power (Westmoreland and Klass, 1990). Arousal is another MM construct. Finally, *distractor suppression*, manifest in the N2pc EP component (Loughnane et al., 2016), aids decision-making. *Distraction* is a further MM construct. MM constructs are, thus, compatible with multiple key ideas of the decision theory. Scalp-EEG correlates (CPP, LRP, N2pc, α, μ-β, θ) encountered in decision research moreover represent electrophysiological evidence for the existence of these MM constructs in the brain.

Some final remarks on thermodynamic cycles and decision-making are as follows. Decision-making is like task completion in the MM Kant Cycle. This Cycle was modeled on the Otto Cycle as the latter entails both generation and dissipation of energy. Improved decision-making may be associated with free-energy optimization in thermodynamic cycles, i.e., minimization of cycle area (Deli et al., 2021). Information (negentropy) flow is equivalent to energy flow since information interconverts with energy (Collel and Fauquet, 2015). Free-energy optimization may therefore also apply to mental analogs of thermodynamic processes like the Kant Cycle. Further, in analyzing brain information flow, the number of synaptic connections plays the role of the thermodynamic mass (Vopson, 2019). The greater the synaptic density, the more information can be obtained from energy. The attribute tree (Figure 2) of consciousness – the contiguity, subordinancy, supraordinancy of coordinate axes – may represent a kind of mind code, a grouping of incoming bits from synaptic connections (from sense organs and elsewhere), that converts information (Shannon negentropy) into concepts with associations. Concepts are the level of behavior, and behavior entails dedicated energy expenditure (and Clausius entropy production), e.g., to contract specific muscles and to recall specific memories. Associations may manifest as connections between concepts that are energetically favorable. Therefore, when one concept is in consciousness, the other pops into consciousness from memory spontaneously. This facilitates the choice of a message out of all possible messages coming out of the synaptic connections. Since local upper and lower energy limits are fixed by entropy transport, shorter paths are thermodynamically favorable. Thus, decision performance may depend on density of synaptic connections and brain networking between regions. Better networking may activate fewer brain modules and links by engaging familiar behavior patterns and automated processes from memory. Other factors biasing the cycle include positive and negative emotions; the latter may reduce decision performance by activating wider brain areas and diverting energy reserves.

## Discussion

### Summary

This manuscript was motivated by the proliferation of (often vague, poorly cross-related) latent psychological constructs in neuroscience. Seeking to mitigate this, we looked to thermodynamics, a field with fewer endpoints, each precisely defined and systematically derived from a few axioms. We attempted to erect a formalism for mental phenomena with the internal consistency (though not yet external validity) of thermodynamics. Guidelines included keeping the number of terms to a minimum and defining derived terms clearly as combinations of a small set of core terms. We wished to produce mental theorems with nontrivial and nonobvious consequences that were empirically testable, i.e., falsifiable. Eventual experimental verification of such derived theorems would speak to the validity of the constructs in which the theorems were phrased. And that could begin a program to thresh out valid from invalid constructs, ultimately aiming at an accurate and definitive formalism. Using the thermodynamic Maxwell Relations as centerpiece, we prepared a detailed analogy – an allegory – between thermodynamic and mental variables. The Maxwell Relations were chosen because they predict outcomes for many scenarios where it is impractical to conduct experiments. These Relations are falsifiable and make nontrivial, nonobvious predictions. In selecting mental variables, we respected the RDoC principle of using only mental constructs that are psychologically valid with a brain basis of some kind.

The result was a mental formalism highly analogous to classical thermodynamics. All mental variables met RDoC (or comparable) criteria. Thereby, most (“time, consciousness, attention,…”) were familiar in neuroscience, although a few (“mental inertia, mental velocity,…”) were semi-endemic to the present framework. The RDoC constructs and units of analysis cited were very general, in some cases consisting of mapping task-related scenarios to large-scale brain divisions. A tighter treatment, for future efforts, would narrow the choices down to effective brain circuits and connectomes. An example of a more exacting treatment would be Damásio (1994), who, in his somatic marker hypothesis, finds “morality” reducible to “long term advantage over short term advantage decisions” and localizes this construct to ventromedial prefrontal cortex. We derived the MMR, stated some of their nonobvious predictions, and suggested how they might be tested in psychological experiments. Analogous to the Otto Cycle, we produced a Kant Cycle using the mental variables. The Kant Cycle illustrates how moment-to-moment conscious experience might evolve from perception to behavior. Using largely introspective methods, we mapped a mental formalism onto classical thermodynamics in high detail. Some of the overlap emerged naturally and unexpectedly. We look forward to expanding the physical analogy, to undertaking point-by-point external validation with prior findings, and to formulating more systematic predictions for future falsification.

There are worthy and widely practiced alternatives to allegory formation, or working with analogies, in constructing theories (Achinstein, 2013). The hypothetico-deductive method is very common, including in neuroscience. In one version of this method, as described by Hempel (1966), investigation is divided into a hypothesis-invention stage and a hypothesis-testing stage. There are no rules for generating the hypothesis, which may simply be a guess or conjecture. The part of logic is limited to drawing deductive inferences from the hypothesis for purposes of experimental testing. In Whewall’s (1840) version of this method, in contrast, reasoning takes on a wider role including the “colligation” of facts in formulating and hypothesis and applying criteria of “consilience” and “coherence” in testing and establishing a theory. In situations where you do not have a theory at all or do not know which experiments to do or you do know which experiments, but they cannot be performed, hypothetico-deductivists like Descartes advise to do nothing – do not speculate. Newton in the Principia, similarly, and famously advised against making hypotheses that are not directly derived from observation, yet he himself made several such hypotheses in the Opticks and in the Principia itself! It is not a priori clear what type of theory best applies to higher mental functions, which experiments would test it, and if those experiments can be performed. Maxwell’s methods making great use of reasoning and analogies are apt for such circumstances, and we opted to use them in developing the present model.

The MM picture is consistent with philosophical interactionism (Popper and Eccles, 1977), the idea that the mental influences the physical and vice versa. When you step from the kitchen into the living room, for example, your consciousness comes along with you and is thereby flooded with fresh sensations. Hence, consciousness is tethered to the body and responds to the physical environment. Most physical events, moreover, take place without human consciousness. We agree with Einstein that the Moon is still there even when we do not look at it (Pais, 1979). On the other hand, physical events also routinely occur with conscious intervention. Standing behind a wheelbarrow, for example, you can image grasping its handles and rolling it 3 m down the road; then you can go and do it, and you can even consciously modify how you are doing it as you do it. The MM formalism allows for physical events occurring outside of consciousness, for physical events influencing mental events, and for mental events influencing physical events, just as we see in everyday life. This formalism proposes that the mental and the physical interdigitate through consciousness serving as an additional indefinable of physics through which the other indefinables can be experienced by humans. The Supplement (8) offers a possible contribution of the MM model to interactionism in the form of a (highly speculative) answer to the question of how the physical and the mental worlds can interact causally.

### Commentary

Why should the mental world resemble the physical? One reason is that both are portrayable as linear-algebraic spaces. Whether attributes of consciousness or physical distances, individual qualities are identified and assigned dimensions. Each dimension has a zero or reference, a maximum or infinity, and a scale or resolution. Mental apprehension and physical measurement both allow arbitrary choice of units. Further, the time dimension is common to mental and physical. The common character of the mental and physical realms as space–times permits the formal description of both with analogous mathematics. As Kant (1781) observed, we experience nothing outside time and space. Or (Campbell, 2001), “When you think about what you have experienced in the apprehension of forms of time and space, you employ the grammar of thought, the ultimate categories of which are: being and nonbeing.” Thus, there are philosophical antecedents to the idea that the physical and mental worlds are both formed of space–time, ultimately divisible into bit-like elements.

Derived from mental or physical space–time is *motion*, change in space per change in time; an object moves across space or the quantity of an attribute in consciousness increases or decreases. Then there is mass or inertia, impediment to motion, the tendency of a concept to linger in consciousness. Working on mass is force, propelling motion in one direction or another; attention pulls concepts into consciousness, holds them against distractors, or pushes them out of consciousness. Finally, electric charge provides an elementary *qualitative* distinction between otherwise identical entities; mentally, an object or concept is recognized as the same or different, as familiar or novel. From these axioms, composite variables are derived and complex phenomena emerge. Thus, parallelism between mental and physical might be expected.

Also relevant are questions of primary data and the unity of mental and physical. If mental and physical worlds are one, they might be expected to engage analogous laws and variables. If the primary data of science constitute subjective mental experiences, it is not surprising that constructs of the physical world are built from elements of essentially mental character. Hence, for various reasons, a high-grade similarity between mental and physical variables is not entirely fortuitous.

Concerning relations between mental and physical, multiple *levels of description* contribute to complexity in the brain. There is a fundamental level of constituent physical particles and their governing laws. Then a structural level, the blueprint of the brain on which form follows function. This may interact with the fundamental level in a manner similar to the influence of initial and boundary conditions on differential equations (holism). Finally, there is the “intentional” level of thought and consciousness, itself capable of acting upon and changing initial and boundary conditions, but without violating some set of fundamental laws properly understood. In the end all is physics, but we appeal to a physics that is not pure materialist, but rather one that explicitly includes the observer, as in von Neumann’s (1932) Orthodox Quantum Mechanics. Integral to orthodox quantum theory is “Process 1,” a free choice by the observer (Stapp, 2007), the experimenter’s choice of which actions to perform. These actions introduce discrete elements by dividing the world into “sample” and “apparatus.” The sample in such experiments acts like a thermodynamic microstate and the apparatus as a macrostate. The handling of the parts of the apparatus as macrosystems in the course of the observer’s freely chosen actions resonates with the notion of a jerk process, like the will, marshaling an ensemble of microscopic particles, by attending to them as a macroscopic object, into a directed behavior. In Orthodox Quantum Mechanics, there is nothing in the physical aspects of nature that determines the choices of Process 1. Hence, quantum mechanics, our most precisely empirically validated scientific theory, postulates an intrinsically mental aspect of reality alongside or subsuming its physical aspect. Here we have explored one notion of a mental world coexisting and (through time) connected with the physical world, whereby both exhibit a parallel form.

### Limitations

Our model does not overcome the usual difficulty of psychological theories, that variables like consciousness, attention,…, are subjective phenomena quantified indirectly through introspective reports, rating scales, and cognitive experimental endpoints. We accept the proposition that the world has inherently subjective aspects. Thus, it may be impossible to get by without introspection completely. Physics, for its part, has historically turned weakness into strength by accepting fundamental limits. The First Law of Thermodynamics meant accepting that we cannot create energy from nothing. The Second Law meant accepting that we cannot convert heat 100% into work. Special Relativity meant accepting that we cannot travel faster than *c*_0_. The Heisenberg Uncertainty Principle meant that the Laplacian dream (or nightmare) of predicting all behavior of a system by knowing all microscopic initial conditions exactly is lost. Yet, each of these limitations ultimately proved empowering and enlightening. We hope that accepting the impossibility of reducing the mind to the four objective indefinables of physics will also advance neuroscience. Doing so need not imply consigning ourselves to the nebulous vagary of those who balk at any hint of imposing form upon the mental domain. Rather we recommend consciousness as a formal fifth indefinable of physics; the conscious observer is the positor of coordinate systems without which measuring other physical variables is impossible. Thus can mental phenomena be integrated into physics. We further indicate time and entropy as points of contact between the physical and mental. With the example of the Maxwell Relations, we suggest patterns along which mental entities may interrelate mathematically in an effort to chart the subjective mental realm systematically and quantitatively.

Finally, as experimentalists, we know how nature routinely frustrates conjecture; why would one expect the present propositions to stand to laboratory verification? Our theory is modeled on classical thermodynamics, the most widely empirically validated of all branches of physics. If we are fortunate, some virtues of this field may accrue to our modest endeavor. Nature, moreover, frequently governs diverse phenomena with comparable mathematical structure. For example, the exponential equations of radioactive decay are similar to those for light transmission, thermal fluctuations, chemical activation energies, etc. In exploring the uncertain territory of higher brain function, it is reasonable to leverage prior successful models. More importantly, our model ventures predictions which are neither obvious nor trivial and which expose it to empirical falsification. We are willing to amend or abandon our theory to the extent disproven by experiment.

## Data Availability Statement

The original contributions presented in the study are included in the article/Supplementary Material, further inquiries can be directed to the corresponding author/s.

## Author Contributions

JON and AS designed the study and wrote the manuscript. Both authors contributed to the article and approved the submitted version.

## Conflict of Interest

The authors declare that the research was conducted in the absence of any commercial or financial relationships that could be construed as a potential conflict of interest.

## Publisher’s Note

All claims expressed in this article are solely those of the authors and do not necessarily represent those of their affiliated organizations, or those of the publisher, the editors and the reviewers. Any product that may be evaluated in this article, or claim that may be made by its manufacturer, is not guaranteed or endorsed by the publisher.
