# Supplementary material for: The Mental Maxwell Relations: A Thermodynamic Allegory for Higher Brain Functions
Source: Front Neurosci. 2022 Feb 28;16:827888. doi: 10.3389/fnins.2022.827888 (PMC8919724; doi:10.3389/fnins.2022.827888)
Supplement: Supplementary file 1 [file Data_Sheet_1.pdf]

frontiers

Supplementary Material

Several matters ancillary to the development of the Mental Maxwell Relations (MMR) in the main text are addressed in this Supplement.

1 Objective Information *vs.* Meaning

We here compare objective information (which we call simply “information”) with *meaning*, which below we will define as “information in transit to behavior”. Information and meaning follow the value/sense distinction from analytic geometry or the value/address distinction from computer science. Consider an 8-bit slide dual in-line package (DIP) switch configured thusly

‗−−‗‗−−−. **8-bit DIP Switch** (S1)

The configuration of the 8 switches, how they are set physically-- which are UP, which DOWN-- is objectively sharable information. It can be perceived equivalently by independent observers without prior agreement. Nor can any observer change the physical configuration merely by thinking about it. That is what is objective about it. The meaning of the configuration, in contrast, requires convention or agreement to be transmitted between observers and depends on the subjective coordinate system assigned to the DIP switch. To understand how a DIP switch can have a coordinate system think of each individual switch as an objective minimalist coordinate axis for which 0 is the origin and the scale and the maximum have been collapsed into 1. *A priori* the 0 and 1 are interchangeable, but subjectively, we can assign them a sense UP or DOWN. Each bit can further be assigned to a different power of 2 so that the 8 bits together form a set of coordinate axes. Meaning depends on such assignments. If, for example, UP means ON and DOWN means OFF, and the switches are numbered with least-significant bit left and most-significant bit right, the configuration shown “means” 11100110 (230 in decimal). But if UP is OFF and DOWN is ON the identical configuration means 00011001 = 25. Wildly different results are obtained from an identical configuration—the same information-- by reversing the sense. And this, and other transformations, can be done subjectively in the mind. The meaning can also be embodied practically by inserting the switch into a printer, fax, etc. The different DIP-switch configurations then regulate the actions of the device, line feed, disconnect, and so on. Meaning is information in transit to behavior. And meaning depends on the *context*, the coordinate system or set of ordinally distinct axes, each holding a number of cardinally indistinguishable bits. In the mind, the MM model sees consciousness (plus memory) as the context, set of axes for the different attributes, and information as the units filling-in those axes. Similar to the settings on DIP switches, sounds, letters, etc. can be recognized (enter consciousness) as concepts without meaning. Once in context, they acquire meaning.

2 Information and Objectivity

How can the mental information variable be objective and, thus, link the mental to the outside physical world? Even the above DIP switches look and feel different to different observers. The answer is that, in discerning objective reality, we normally adjust our bare perceptions. For example, gazing at a distant tree behind a nearby horse, the tree appears smaller, but in reality is bigger. One learns to compensate for this in the mind, to adjust for distance and other factors. Objective reality dwells on the far side of those adjustments. Adjustments are made physically, e.g., walking to the tree, wearing corrective lenses to view the DIP switch, when practical, but are more often performed mentally. One does not walk up to the trees on the distant mountainside, one glances at them and knows they are taller than they appear. And observing any physical phenomenon there is typically a residue of observed properties corrected for only mentally, if at all. The paradox emerges that objective reality exists only as an abstraction, an ideal, what is never observed directly but what *would* exist if all the necessary corrections were applied. This is another reason why mental variables are required for a complete picture of the world. One way out of this paradox, might be to define the objective state of a phenomenon as the set of attribute values resulting from a limiting process (for all relevant adjustments) across the set of all distinct observers. This would be the objectivity we work with practically and scientifically. Mental information in the MM model, however approximately, has this character.

Objective information is measured in bits along subjective attribute axes. Since, as mentioned, a bit is itself a coordinate axis in limit form where ∞ and 1 collapse into a single pole, this implies that, in the limit, meaning becomes objective, it becomes information. One bit is the *lower limit* of knowledge. Information is communicable because every human being shares this lower limit. One cannot communicate *less* than a 0 or a 1 on a *single* axis. One can do no less than recognize that an object or idea has or has not one bit of an attribute. For all who share this lowest common denominator, it is a basis for communication, for objectivity. Objectivity is possible when different observers have common limits of perception. I.e., different observers arrive at the same count—and therefore share information—because they neglect fine detail at a common resolution. This potential to share information renders information objective. Alternatively, a bit is the smallest distinguishable element of conception or perception. A bit on an attribute scale is either there or not there. One is not conscious of resolution finer than the bit nor of distinctions between adjacent bits on an attribute axis. Thus, bits behave like pure numbers. And consciousness is the positing of distinctions between blocks of information.

3. Unreasonable Effectiveness of Mathematics

Wigner (1960) famously wrote about "the unreasonable effectiveness of mathematics in the natural sciences". This effectiveness may in part be due to the flexibility of mathematics in working at any level of approximation and in dealing with facts both in evidence and not in evidence. It may also

have to do with the above-mentioned fundamental role of the bit in perception. Physics is concerned with the perceivable and the objectively communicable. These are ultimately expressible in bit-form. Bits are the basis of numbers and numbers are the basis of mathematics. It is therefore reasonable that mathematical formulae accurately and precisely denote our experiences and convey them objectively to others. Even phenomena too small or too large, too fast or too slow to be perceived directly must, at least indirectly, e.g., thru readings on instruments, be rendered observable in the field or in the laboratory. Each bit is a tiny attribute field of “Yes” or “No’, which either overlaps or does not overlap accurately with the objectively transmissible picture of the physical scene. During analysis, one does not look inside the bit-field, one treats it as homogeneous. Separate bits are treated as indistinguishable, apart from their arbitrary external addresses. This gives bits the properties of numbers. It implies that, at a given resolution, the bit-mapped scene obeys all the theorems, laws, etc. derivable for numbers. That is why mathematics describes Nature as well as it does.

4 The Cartesian Theater and the Homunculus Argument

Theories like MM that advocate space-like consciousness are occasionally berated as Cartesian Theater models and are critiqued based on the Homunculus Argument. Consciousness is described as an image of the world projected onto a screen watched by a little man (the homunculus) inside the Cartesian Theater of the brain. The problem is that there must be another screen inside the little man and another and another in an infinite regress. Thus, space-like models of consciousness would be destroyed by logic. Fortunately, there is a way out of this dilemma. A similar situation occurs in general relativistic cosmology. The Universe is said to have a curved geometry of finite size. An obvious question is, must there not some larger, Euclidean space, in which this curved universe is embedded? The answer is, no. Gauss’s *Theorema egregium* states that the curvature of any surface (and even an N- dimensional space like MM consciousness qualifies as a kind of surface) is fully determined by its *internal* properties. No reference need be made to the space in which the surface is embedded, in fact, this space need not even exist. An analogous situation applies for the Cartesian Theater in which each homunculus embeds all the larger homunculi above him. The *Theorema egregium* implies that none of the homunculi need exist. Consciousness can “ground-out” as a surface (space-like entity) on a single level.

5 Concrete and Abstract Concepts

The MM notion of consciousness as a phase-space enables the formalism to accommodate both *concrete* and *abstract* concepts. For concrete concepts, like the leaf example used in (3.3.1), attributes predominate over associations; for abstract concepts, associations predominate over attributes. Thus, for example, for a concrete concept like an apple, many sensory attributes are readily manifest in mind (redness, roundness, hardness, gloss, smoothness, heft,…), while for an abstract concept like “administration” there are few (perhaps the face of a clerk, the image of a blank form, etc.) The concept “administration”, however, has many associations and is evoked by and evokes numerous other concepts in text or speech, e.g., “policy”, “protocol”, “scheduling”, “management”,

"leadership",… To a degree abstract concepts, especially imprecisely defined ones, survive by virtue of their frequent repetition, particularly in the company of other abstract concepts. This occurs to the point where these concepts sometimes enjoy only a tenuous anchoring to any concrete meaning and our discourse risks sinking into a pure verbalism of one abstract concept attached to another.

6 Syntactic Potential

In our model, syntactic roles or categories (subject, direct object, etc.) are analogous to *chemical species*. Our mental analogue for the thermodynamic chemical potential is the "syntactic potential". The syntactic potential of syntactic category i is the internal information transferred into the task per concept of category i introduced into (or taken out of) the task. For example, let the task be forming a sentence, beginning with one word:

"He…"

This pronoun invites enormous semantic possibilities in a vast memory network having a certain value of internal information. We add a word:

"He walks…"

The added concept is in a new syntactic category, verb. Intersection with another memory network for the new word restricts the field of semantic possibilities, modifying the internal information of the sentence. The syntactic potential here is the rate of change of internal information with respect to adding one verb, whereby the numbers of concepts in the sentence that are subjects (one), direct objects (zero) and all other categories (zero) remain constant, along with consciousness, arousal, etc. A third word

"He walks her…"

is a direct object. The appropriate syntactic potential is the rate of change of internal information with one direct object added, other quantities remaining constant. One adds further words

“He walks her hastily...”

“He walks her hastily outside.”

to finish the sentence, inducing further syntactic potentials for adverbs. Hence, we are discussing how the memory information accessed is affected by introducing (or removing) one or more concepts with varying syntactic roles into a task or sentence. As a motor task example, one might start by uplifting the right hand, add a hammer, add a nail, start striking, and then take-on a tempo for striking, then determine how much information is added at each step. The units of syntactic potential are bit/concept. Syntactic structures are included in RDoC under Language Behavior. Syntax processing has traditionally been localized to peri-Sylvian (inferior frontal, superior temporal and middle temporal) cortices (Grodzinsky & Friederici, 2006; Friederici & Weissenborn, 2007).

7 A Detailed Example for Experimental Testing of the MMR

Let us consider one way one might test the first MMR in the main text Eq. (7)

$$\left(\frac{\partial\{\text{arousal}\}}{\partial\{\text{consc deform}\}}\right)_{\{\text{distraction}\}} = -\left(\frac{\partial\{\text{salience}\}}{\partial\{\text{distraction}\}}\right)_{\{\text{consc deform}\}}. \tag{S2}$$

This MMR states that the rate-of-change of arousal with respect to consciousness deformation at constant distraction is equal to minus the rate-of-change of saliency with respect to distraction at constant consciousness deformation. Experimental validation would consist of measuring these two rates and determining if they really are equal to each other (within error). To achieve that, we need to measure arousal, consciousness deformation, distraction, and saliency somehow. Moreover, we will best measure them in a “meditative state”, our mental correlate of thermodynamic equilibrium, when the Maxwell Relations are valid.

To test this MMR, you would take an experimental subject performing a cognitive task and run a series of trials in which you vary, say, the saliency of the task target stimulus and the level of distraction and measure, say, the subject’s level of arousal and consciousness (see below). When you are done, you plot the values and map out a surface in a saliency-consciousness deformation-arousal-distraction space (analogous to a PVTS-diagram in thermodynamic state space). You identify the curves where this surface intersects planes of constant distraction (analogous to isentropic planes) and measure the slopes Δarousal/Δ(consc deform) along these curves. Then you identify the curves formed by intersection of the surface with planes of constant consciousness (analogous to isochoric planes) and measure the slopes Δsaliency/Δdistraction along these curves. Finally, you determine whether these two sets of curves have opposite slope (Δarousal/Δ(consc deform) = -

Δsaliency/Δdistraction) wherever they intersect. So we are actually testing a question about the topology of the mental state space.

Here is one of many paradigms feasible with existing cognitive psychological techniques or modest extensions thereof. The point here is not to critique the paradigm *per se*, but rather to illustrate that the prediction might be tested by some stretch of the imagination, without invoking pie-in-the-sky methodology. You have a test subject lie alone supine on a cot in a darkened room. He or she wears video goggles and sound-attenuating headphones. You might tape the fingertips to minimize tactile sensation. He or she wears a standardized exam gown and you allow him or her time to habituate to the tactile sensations of the gown and cot. You could even do the experiment in a sensory-deprivation tank if you want to be very rigorous. One or two EEG leads are attached to the scalp. Thru the goggles, the subject is presented with a red fixation cross on a background in the center of the visual field. The subject is given the task of focusing on the "red" of the cross—and nothing else. He or she is instructed to shift gently back to the red if his or her attention slips away to anything else, and to repeat this no matter how many times it happens. This is intended to induce a meditative state; again, if you want to be very rigorous, you can use a trained meditator as subject. The subject is allowed to lie for a while as described. We conduct several trials of this of fixed duration, whereby we vary the experimental parameters from trial-to-trial. In particular, we vary the redness of the cross across higher and lower degrees of saturation. We vary the background from plain white or plain black, adding colors, adding textures, etc. We can do the same within the body of the cross. (Remember, the task is to focus not on the cross *per se*, but on the redness of the cross.) In particular, we can vary the saliency of this redness, e.g., whether the cross is uniformly red against a stark uniform white background, whether the cross is mottled with red plus other colors and textures, possibly against a mottled background, etc. Finally, we occasionally introduce "distractors" into the scene, e.g., gentle flashing lights, auditory tones, or mild electroshocks. All of this is standard psychophysiology methodology, well within the experimental cognitive psychologist's toolbox.

The EEG is used as a proxy for "arousal", we examine the record for the intrusion of sleep waveforms. This can also be calibrated separately offline for the individual subject, comparing clinical ratings of wakefulness to the EEG power spectrum. The MMR formalism measures arousal in "ºGCS" (Glasgow Coma Scale), but other units might do as well.

"Consciousness" will be measured as the number of "attributes", i.e., the number of independent qualities experienced by the subject. "Consciousness deformation" would be the fractional change in this number, relative perhaps to a pre-task baseline. The purpose of all the sensory attenuation is to keep this number down to a manageable level. As we insert dimensions (e.g., different colors, grainy textures, curves or sharp edges, etc.) into the stimulus and background presentation, we presume that we are adding dimensions to the subject's momentary conscious experience. As the scenario is, by design, simple and boring the subject will, in fact, be more inclined to be conscious of each available dimension. But to be more sure, we can query the subject with a series of yes/no questions after each trial: "Did you see red? Did you see green? Did you see something sharp? Did you see something round?,..." each answered by quietly pressing a key on a response box. If you want to be more rigorous, you could even query about intrusions of thoughts, memories, and fantasies during each trial, recording the subject's report in as much detail as he or she can give. It might help there to have an articulate subject. In the end, you tally-up the number of independent attributes and divide by the reference number for your metric of consciousness deformation.

In the MMR formalism, "saliency" is measured in units of bit/attribute^N. We will quantify the information content of the attribute focused upon, in this case the red of the cross. Again this can be calibrated to the individual subject separately offline. We determine the subject's discrimination threshold (resolution) between two different shades of red. Then we determine the full range of red that he or she can perceive, from zero to maximum saturation. The resolution becomes our unit for gauging the information content, the amount of red in the red cross (how many of those minimum discernable degrees of difference are present?). This metric divided by the number of attributes in the scene to the Nth power is the saliency endpoint.

Finally, "distraction" in the MMR formalism is measured in units of bit/°GCS (analogous to entropy in J/K). We again assay the information content (across all attributes) of the distractor stimulus used in the trial. Again, this can be based on offline calibration. The distractor information content divided by the arousal level is the distraction endpoint.

One tabulates arousal, consciousness deformation, saliency, and distraction for various combinations of parameters across many trials. One picks out a subset of trials where distraction was approximately constant and calculates Δarousal/Δ(cons deform) for these trials; one picks a subset of trials where consciousness deformation was approximately constant; -Δsaliency/Δdistraction is calculated for these trials. Then one sees if these two pairs of ratios are approximately equal. If so, the first MMR is validated; if not, it is invalidated. The procedure is complicated, but no more so than many cognitive experiments being performed routinely in labs all over the world.

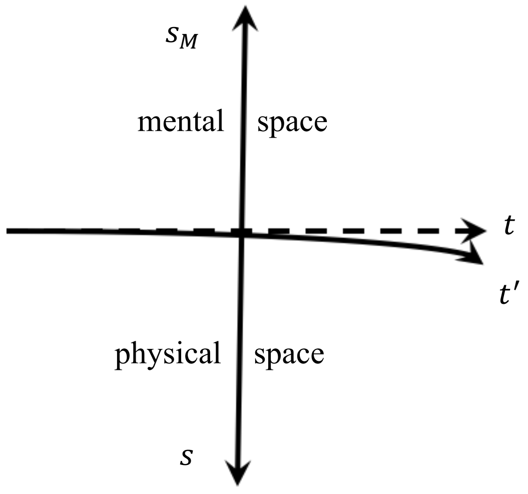

FIGURE S1. In the Mental Maxwell model, the time axis (horizontal) is a border shared by the mental (*upper*) and physical (*lower*) realms. Mental (s_M; *upper*) and physical (s; *lower*) space are on the vertical axis. Any distortion of mental time, i.e., any transformation $t \to t'$, occasioned by a process in mental space would manifest in physical space as well, presumably just locally within the brain. The time-shift could change the rate of force production, $\Delta Y = \frac{dF}{dt} - \frac{dF}{dt'}$, in physical space as well, thus resulting in causal efficacy of mental action. The physical realm could affect the mental realm by the same process in reverse. This is one (highly speculative) mechanism for philosophical interactionism.

8 Speculative Mechanism For Mental-Physical Interaction

The main text (3.2) indicates that the variable time is both mental and physical, although mental time is distorted relative to physical time. One can, therefore, view the time axis as a border between the mental and physical realms, as in Figure S1. If the time axis is shared between mental and physical then any distortion in mental time will manifest, at least locally within the brain, in a distortion of

physical time as well. Physical time will be transformed $\mathrm{t} \rightarrow \mathrm{t}'$. That implies that the physical yank in that local brain region will also be transformed

$$Y = \frac{dF}{dt} \rightarrow Y' = \frac{dF}{dt'}. \tag{S3}$$

and the difference $\Delta\mathrm{Y} = \mathrm{Y}' - \mathrm{Y}$ would be the rate of mental force production (or consumption) acting on the physical world. Other quantities that are functions of time, including force itself, would be changed as well, but we focus on the yank because will and emotions are the MM correlates of the yank (3.4.6). Hence, mental exertions of will or outbursts of emotion might manifest synchronously in the physical world (ultimately in muscle contractions and relaxations) in this way. The same action in reverse would allow the physical to affect the mental. This is a highly speculative potential mechanism for philosophical interactionism.

How could such mental time distortion arise? In Special Relativity, one kind of distortion, time dilation, arises because the vacuum (and all material media) have non-zero electromagnetic reactance. This implies that they slow down the rate of information transfer to a maximum of c_0. This slowing leads to length contraction, mass increase, and time dilation. A very concrete force, magnetic force, is produced because electric charge is unaffected by these relativistic changes. The relativistic effects mutually manifested by two systems moving in different frames-of-reference may also be thought of holistically, i.e., as consequences of the properties of the enveloping void that separates (and connects) them both. This perspective is reminiscent of the lumineferous aether the existence of which, per common interpretation, was obviated with the ascendance of Special Relativity. But Maxwell (1873) cherished his aether, which Wilczek (1999) has recently attempted to revive. Perhaps a mental slowing of informtion transfer could produce force as well.

The brain exhibits reactances well beyond the electromagnetic kind. It filters inputs heavily, witholds responses pending decision-making and conflict-resolution, and, thru various types of memory, expresses acute to very long-term delayed influences. The brain, like any organ, strives for homeostasis, i.e., to maintain operating parameters like body temperature, blood pH, etc., constant independent of what happens in the outside world. It succeeds at that, granted that the outside world provides nutrients and oxygen, is not overly harsh, etc. The brain is a very complex medium. It is a colloid with numerous lipid membranes separating aqueous phases in extremely complicated geometry. Therefore, surface and electrochemistry are involved. Many, many chemical species inhabit these phases, so brain tissues may approximate infinite-component solutions. Such complexity may enable complex dynamics, whereby one input state can lead to many different output states or, alternatively, the same output state can be reached via numerous different paths. These dynamics and the uncertainties they engender may provide a space for the emergence of novel phenomena. With its high degree of connectivity the brain is a system that interacts more with itself than with the outside world. With all these factors the brain enjoys a degree of autonomy from the outside world. Perhaps this is what makes it into a harbor for consciousness, a phenomenon that operates in part independent of the environment, and for agency, on some occasions when it does interact with the environment. Mental constructs like volition fall into the category of non-random and non-determined. On the example of neutron diffraction by crystals, Rauch (1995) demonstrated

that phenomena exist in nature that are neither stochastic nor deterministic. Perhaps the brain is a further milieu that can spawn such effects. Finally, the autonomy of the brain may result, at least for certain processes, in the brain running its own clock, leading to a distortion of mental time relative to objective laboratory time.

The influences of the mental realm upon the physical may also be holistic in character. Holism is pervasive but understated in mainstream physical science. Take a typical chemical reaction, for example the double replacement

$$CaCl_{2(aq)} + Na_2CO_{3(aq)} \Leftrightarrow CaCO_{3(s)} + 2NaCl_{(aq)}. \quad \text{(S4)}$$

The subscripts "(aq)" indicate that $CaCl_2$, Na_2CO_3, and NaCl are not present as whole molecules in empty space, but rather as dissolved ions in an aqueous medium. The medium is the holistic part; it contributes to and, in fact, enables the reaction. Similarly, the subscript "(s)" indicates that the $CaCO_3$ is present as a (in this case crystalline) solid, another form of holistic medium that impacts the reaction. Written reactions like this are characteristically festooned with a variety of symbols $(\Delta, h\nu, pH7, \cdots)$ pointing to a larger environment that participates in the reaction beyond the principal reactant particles. In depicting chemical reactions and other thermodynamic processes, one frequently makes use of heat sinks and reservoirs, concentration reservoirs for diffusion, and the like. These are further holistic entities that intrude into mechanistic treatments. Critical mass in nuclear fission and other reactions is a further example; the scale of the macroscopic reaction ensemble has a decisive influence on the rate and course of the process at the microscopic level. In classical mechanics, it is very common to draw free-body diagrams that feature, for example, a weight on a spring hanging from a beam or a weight lying on a floor. Beam and floor are typically assumed immovable and denoted by diagonal hatching. These are the holistic elements of such diagrams, for the beam is presumably attached to a ceiling, thence to a building that sits on a foundation and so on to the wider world. The same applies to the floor. A further holistic element is the gravitational field causing the weights to hang or to rest on the floor. An elementary particle reaction in vacuo, such as neutron decay into a negative pi-meson

$$n \rightarrow p + \pi^{-} \quad \text{(S5)}$$

might be thought of a pure reductionistic interaction, free of environmental influences. But the very assumption of a vacuum implies an environment of a very special kind. One must employ a turbomoelcular pump or other means to achieve high rarefaction, one must shield against cosmic rays, one must isolate from electromagnetic fields, etc. For the norm in nature is that there are many particles of different kinds buzzing by, there are cosmic rays passing thru, there are ambient fields, and so on. The maintenance of the vacuum implies a larger suprastructure and therefore holism. The influence of holistic features is frequently not explicit but expressed as initial or boundary conditions. Mental influences within the brain may similarly act diffusely by setting or altering the conditions for

physical processes. Pribram (2013) has long argued for “brain fields” or other holistic top-down effects as organizing principles for higher mental functions.

9 Supplementary References

Friederici, A.D., Weissenborn, J. (2007). Mapping sentence form onto meaning: The syntax–semantic interface. *Brain Res.* 1146, 50-58.

Grodzinsky, Y., Friederici, A.D. (2006). Neuroimaging of syntax and syntactic processing. *Curr. Opin. Neurobiol.* 16, 240-246.

Maxwell, J.C. (1873). On action at a distance. *Nature* 174(7),323-325; 175(7),341-343.

Pribram, K.H. (2013). The Form Within: My Point of View. Westport, CT: Prospecta Press.

Rauch, H. (1995). More quantum information due to postselection in neutron interferometry. Ann NYAS 755, 263-287.

Wigner, E. (1960). The unreasonable effectiveness of mathematics in the natural sciences. *Comm. Pure Appl. Math.* 13 (1), 1-14.

Wilczek, F. (1999). The persistence of ether. *Physics Today* 52(1),11-13.
